# The *Aeromonas salmonicida* subsp. *salmonicida* exoproteome: determination of the complete repertoire of Type-Three Secretion System effectors and identification of other virulence factors

**DOI:** 10.1186/1477-5956-11-42

**Published:** 2013-09-27

**Authors:** Philippe Vanden Bergh, Manfred Heller, Sophie Braga-Lagache, Joachim Frey

**Affiliations:** 1 Institute of Veterinary Bacteriology, University of Bern, Länggassstrasse 122, Bern, Switzerland; 2 Department of Clinical Research, University of Bern, P.O. Box 37, 3010, Bern, Switzerland

## Abstract

**Background:**

*Aeromonas salmonicida* subsp. *salmonicida*, the etiologic agent of furunculosis, is a major pathogen of fisheries worldwide. Several virulence factors have been described, but the type-three secretion system (T3SS) is recognized as having a major effect on virulence by injecting effectors directly into fish cells. In this study we used high-throughput proteomics to display the differences between in vitro secretome of *A. salmonicida* wild-type (wt, hypervirulent, JF2267) and T3SS-deficient (isogenic *ΔascV*, extremely low-virulent, JF2747) strains in exponential and stationary phases of growth.

**Results:**

Results confirmed the secretion of effectors AopH, AexT, AopP and AopO via T3SS, and for the first time demonstrated the impact of T3SS in secretion of Ati2, AopN and ExsE that are known as effectors in other pathogens. Translocators, needle subunits, Ati1, and AscX were also secreted in supernatants (SNs) dependent on T3SS. AopH, Ati2, AexT, AopB and AopD were in the top seven most abundant excreted proteins. EF-G, EF-Tu, DnaK, HtpG, PNPase, PepN and MdeA were moderately secreted in wt SNs and predicted to be putative T3 effectors by bioinformatics. Pta and ASA_P5G088 were increased in wt SNs and T3-associated in other bacteria. Ten conserved cytoplasmic proteins were more abundant in wt SNs than in the *ΔascV* mutant, but without any clear association to a secretion system. T1-secreted proteins were predominantly found in wt SNs: OmpAI, OmpK40, DegQ, insulinase ASA_0716, hypothetical ASA_0852 and ASA_3619. Presence of T3SS components in pellets was clearly decreased by *ascV* deletion, while no impact was observed on T1- and T2SS. Our results demonstrated that the *ΔascV* mutant strain excreted well-described (VapA, AerA, AerB, GCAT, Pla1, PlaC, TagA, Ahe2, GbpA and enolase) and yet uncharacterized potential toxins, adhesins and enzymes as much as or even more than the wt strain. Other putative important virulence factors were not detected.

**Conclusions:**

We demonstrated the whole in vitro secretome and T3SS repertoire of hypervirulent *A. salmonicida*. Several toxins, adhesins and enzymes that are not part of the T3SS secretome were secreted to a higher extent in the extremely low-virulent *ΔascV* mutant. All together, our results show the high importance of an intact T3SS to initiate the furunculosis and offer new information about the pathogenesis.

## Background

*Aeromonas salmonicida* subsp. *salmonicida*, a gram-negative bacterium, is the etiologic agent of furunculosis, a frequent and major pathogen of fisheries worldwide which is generating significant economic losses related to deficits in zootechnical profits and the intensive use of antibiotics [1]. To date, several virulence factors have been characterized for *A*. *salmonicida*: the type three secretion system (T3SS) encoded on a large plasmid and described for the first time in the *Aeromonas* genus in our laboratory ten years ago [2,3]; the surface layer protein VapA [4]; a type I pilus [5]; three type IV pilus systems [6,7]; superoxide dismutases [8] and some extracellular proteins including serine protease (AspA) [9], glycerophospholipid:cholesterol acyltransferase (GCAT or SatA) [9,10] and several hemolysins (aerolysins) [11]. Other putative virulence factors were identified without experimental evidence [12]. However, the T3SS is the only one recognized as having a major effect on virulence, as independent studies have shown that isogenic mutant strains for T3SS structural proteins are non-virulent both in vitro and in vivo [2,13-16]. T3SS is also called “injectisome” because it enables the secretion and simultaneous injection of effector proteins produced in the prokaryotic cytoplasm across the bacterial envelope and then, through a needle and a translocon complex, into the target eukaryotic cells across their membrane [17]. Once injected in the eukaryotic cytosol, effector proteins are able to modulate cell signalling pathways, or alternatively disrupt the dynamics of the cytoskeleton, thereby modulating host cell biology for the benefit of the pathogen [17].

Currently, four different virulent effectors have been investigated for the *A. salmonicida* T3SS, and only two have been studied in detail: the bifunctional toxin AexT, which possesses a GTPase-activating domain acting on small monomeric GTPases of the Rho family and an ADP-ribosylating domain, which ADP-ribosylates both muscular and non-muscular actin [18,19]; AopP, which inhibits the NF-*κ*B signaling pathway by preventing translocation of NF-*k*B into the nucleus of the target cells [20]. AopO, which is related to *Yersinia* YopO/YpkA [14] and AopH with similarity to *Yersinia* YopH [14], represent two further potential effectors that have been characterized in less detail. AexT, AopO and AopH toxins do not seem to be solely responsible for *Aeromonas* virulence because individual knock-out mutations of these genes [14] or a triple-effector knock-out mutant [21] keep a virulent phenotype or show only delayed virulence, such as in the case of *ΔaexT* mutants [14,19]. Given that *A*. *salmonicida* mutants that are defective for T3SS fully lose their pathogenicity, we hypothesize that other important cytotoxic proteins might be injected by these *Aeromonas* nanosyringes into the fish cell cytoplasm.

The aim of this work was to use high-throughput proteomics to display the secretome of *A. salmonicida* subsp. *salmonicida* wild-type (wt, hypervirulent) and an isogenic T3SS-deficient mutant (*ΔascV*, extremely low-virulent) during the exponential-growth phase (GP) and the stationary phase (SP). In this article, which is the second part of the work, authors characterized the whole in vitro repertoire of T3SS effectors and new virulence factors of *A. salmonicida*. In the first part, “*The Aeromonas salmonicida subsp. salmonicida exoproteome: global analysis, moonlighting proteins and putative antigens for vaccination against furunculosis*”, the same authors focused on the general analysis of proteomics data, the presence of cytoplasmic proteins with putative moonlighting activities in supernatants and the identification of putative antigens for fish vaccination against furunculosis.

## Results and discussion

### *A. salmonicida* T3SS and comparison to other appendages

*A. salmonicida* subsp. *salmonicida* wt strain was previously shown to cause 80% - 100% mortality in rainbow trout at 500 cfu inoculated intraperitoneally, while the *ΔascV* deletion mutant derived thereof was shown to be non-virulent causing 0% mortality under the same conditions [15,22]. In order to further show the strong attenuation due to the *ΔascV* deletion mutation, rainbow trout kept under the same conditions were challenged intraperitoneally with 10^8^ cfu, an infectious dose which is not representative of what happens in natural infection. These fish showed only a slight mortality of 20% after 14 days post infection showing the high degree of attenuation obtained with the *ΔascV* mutation. We assume that the residual mortality observed in this experiment is solely due to the excessive load of bacteria applied.

We identified a total of 2136 *A. salmonicida* proteins with PMSS and LFQ values among the different experimental conditions (see Methods for explanations and the first part of this work for raw data) for 1861 and 2070 proteins respectively. These values correspond to a semi-quantitative abundance estimate of protein species present in SDS-PAGE gels and were used as a surrogate for the amount of secreted proteins in concentrated SNs and the amount of produced proteins in whole pellets.

**Figure 1:**
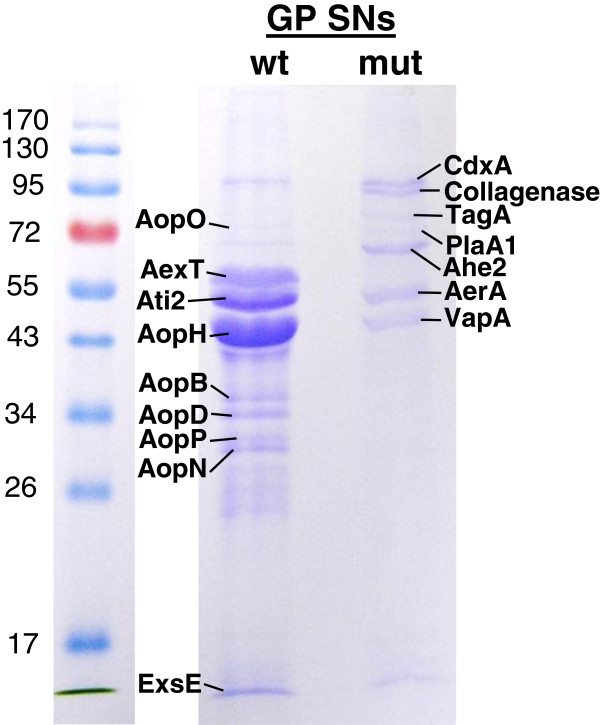
**SDS gel electrophoresis of *A. salmonicida *subsp. *salmonicida *proteins from GP wt and***Δ****ascV *mutant SNs stained with Coomassie blue.** SDS gel electrophoresis of proteins from supernatants (SN) of wild-type (wt) and *ΔascV* mutant (mut) strains in exponential (GP) phase of growth. Proteins corresponding to the most abundant bands are indicated. The molecular weights (kDa) of the Protein Ladder are shown on both sides of the figure.

**Figure 2:**
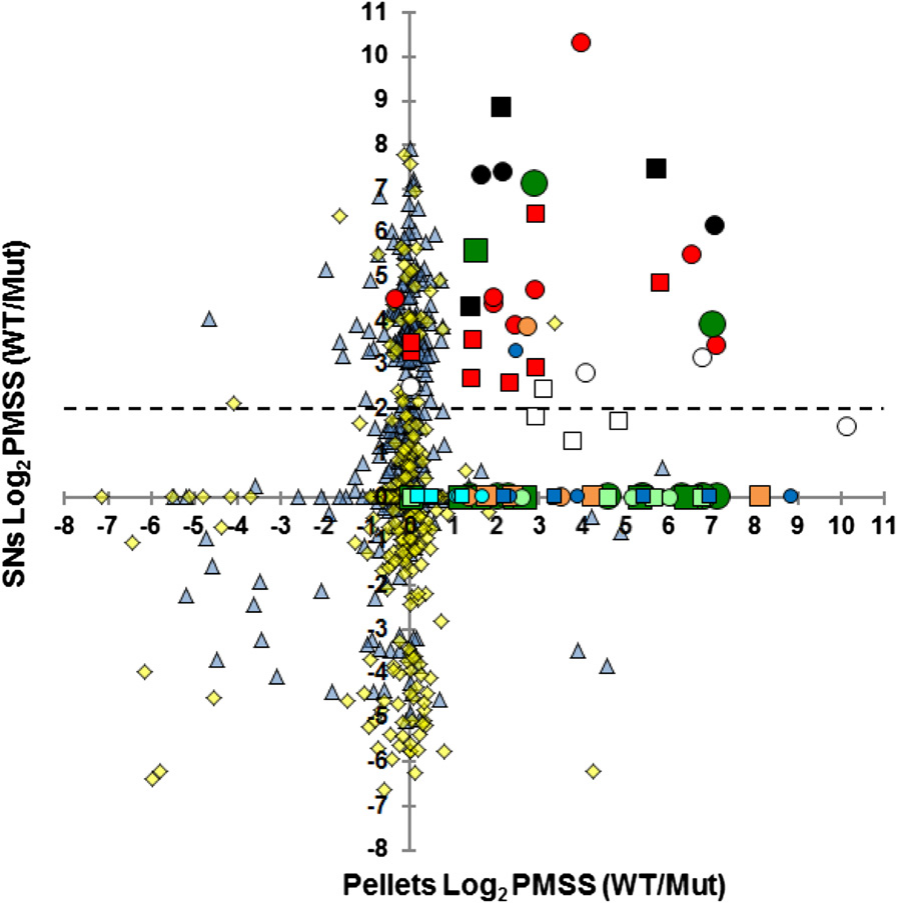
**Ratios of different T3SS components in *A. salmonicida *wt versus mutant strain.** The plot represents the logarithm base two of the ratios [wt/mut] PMSS values for each protein identified in pellet (X-axis) and SNs (Y-axis). For T3SS components, exponential growth phase (GP) values are represented by circles and stationary phase (SP) values by squares. Red, the T3SS effectors; dark green, T3SS chaperones; white, translocators; black, needle components; orange, OM secretin ring; light green, IM export ring; dark blue, C ring ATPase; light blue, transcriptional regulators. Values of other proteins in GP and SP are shown with dark blue triangles and yellow squares respectively. The hatched line represents the threshold that we used to identify proteins 4-fold more present in wt SNs.

In our MS analysis we identified 45 proteins of the *A. salmonicida* T3SS. The effectors should only be secreted or detected in higher quantity in wt SNs (in GP and SP) in comparison to the *ΔascV* mutant (Table 1). Our results confirmed the secretion of the well-described AopH, AexT, AopP and AopO effectors (Figure 1, Table 1). Moreover, we demonstrated the secretion of additional T3SS effectors for the first time. Ati2 (ASA_P5G045), an inositol polyphosphate 5-phosphatase already described as a putative T3SS effector [12], was strongly secreted in wt SNs (as much as AexT, 20 times more than in *ΔascV* mutant SN). Ati2 is homologous to the *Vibrio parahaemolyticus* T3SS effector VPA0450 and *Photorhabdus luminescens* Plu4615 (87% identity over 495 amino acids [aa]). This effector disrupts cytoskeletal binding sites on the inner surface of host membranes, causes plasma membrane blebbing and probably contributes to cell death by facilitating lysis [23]. Our data showed that Ati1 (ASA_P5G046), the chaperone of Ati2, was also secreted in wt SNs by the T3SS, whereas all other T3SS chaperones (SycE, SycH, SycO, AscY, Acr1, Acr2, AscB, AcrG, AscG, AscE, AscO and AcrR) were only present in pellets and were never secreted (Figure 2) suggesting that Ati1 might be injected with Ati2 into fish cells. AopN (ASA_P5G075) was secreted by the T3SS in wt SNs, but to a lower extent than the previous effectors. AopN homologues in other bacteria are T3SS effectors which play a role in virulence and can have a dual role: controlling the secretion of translocator proteins inside bacteria and suppressing immunity when T3 translocated inside host cells [24-26].

**Table 1:** **Secreted components of the *A. salmonicida *T3SS**

**Locus**	**Uniprot**	**Name**	**Description**	**Fold change in SNs Log**_**2 **_**wt/mut [PMSS]**^**1 **^**and [LFQ]**^**2**^		**[Unique peptides]**^**3 **^**and [MS/MS Count]**^**4**^				**PEP GP**	**PEP SP**	**Modlab (>0.4)**	**Effective (>0.95)**	**SignalP**	**TatP**	**Eukaryotic domain**
				**GP**	**SP**	**wt GP**	**mut GP**	**wt SP**	**mut SP**							
**Effectors**																
ASA_P5G009	G7D171	AopH	Putative tyrosine phosphatase	4.06^**1**^	3.07	62^**3**^	37	67	61	0.00E + 00	0.00E + 00	n	y	n	n	PF00102
				9.36^**2**^	8.25	2753^**4**^	288	7348	2155							
ASA_P5G045	A4SUE7	Ati2	Inositol polyphosphate 5-phosphatase	4.41	3.61	60	41	51	46	0.00E + 00	0.00E + 00	y	y	n	n	PF03372
				6.78	9.27	3078	339	4161	521							
ASA_4266	G7D0E2	AexT	GTPase, ADPribosylase	3.91	2.99	51	34	45	43	0.00E + 00	0.00E + 00	y	y	n	n	PF01129
				7.78	9.09	2178	315	4530	708							
pAsal1_03	G7D197	AopP	Inhibition of NF-*k*B translocation in nucleus	3.46	3.34	27	14	23	19	4.12E-284	0.00E + 00	y	y	n	n	-
				5.01	7.46	281	70	138	18							
ASA_P5G098	G7D0W5	AopO	Putative serine/threonine kinase	10.34	6.46	40	7	53	33	5.48E-235	0.00E + 00	y	y	n	n	cd06612
				11.93	12.38	94	0	483	4							
ASA_P5G075	A4SUH7	AopN	Secretion control of translocators and immune suppressor	4.53	2.72	17	10	23	19	1.69E-209	0.00E + 00	y	n	n	n	-
				6.82	6.70	178	22	455	73							
ASA_P5G062	A4SUG4	ExsE	Regulator, chaperone for ExsC	4.52	3.52	9	4	10	9	2.73E-106	2.98E-113	n	n	n	n	-
				5.85	8.56	51	7	101	37							
**Translocon**																
ASA_P5G065	A4SUG7	AopB	Translocon, Hydrophobic translocators, Pore in host cell	6.77	2.88	21	11	16	13	0.00E + 00	0.00E + 00	y	y	n	n	-
				11.12	6.11	428	16	381	34							
ASA_P5G064	A4SUG6	AopD	Translocon, Hydrophobic translocators, Pore in host cell	4.06	3.07	24	13	31	26	0.00E + 00	0.00E + 00	y	y	n	n	-
				7.59	8.98	322	26	1377	158							
ASA_P5G067	A4SUG9	AcrV	Middle substrate, tip translocon, Hydrophilic translocators, Protective antigen, anti-host factor	10.13	3.77	28	8	30	26	6.61E-233	0.00E + 00	n	y	n	n	-
				10.54	9.59	85	0	391	50							
ASA_P5G066	A4SUG8	AcrH	Chaperone for AopB/AopD	0	4.81	3	2	5	3	9.26E-13	5.80E-22	n	n	n	n	PF13414
				6.30	4.94	7	1	10	1							
**Needle**																
ASA_P5G054	A4SUF6	AscF	Early substrate, needle subunit	6.19	7.48	7	3	9	6	1.26E-105	9.02E-226	n	y	n	n	-
				4.07	8.05	12	0	46	0							
					
ASA_P5G078	A4SUI0	AscP	Needle length control, Ruler protein, Regulation of secretion, substrate specifity switch	7.41	4.32	7	1	14	9	3.21E-68	0.00E + 00	y	y	n	n	-
				24.39	10.76	28	0	87	4							
ASA_P5G052	A4SUF4	AscH	Regulator needle assembling	7.35	8.85	12	0	19	14	7.89E-31	1.86E-101	y	n	n	n	-
				25.77	8.04	17	0	100	6							
**Others**																
ASA_P5G046	A4SUE8	Ati1	Chaperone Ati2	7.11	5.62	7	2	3	1	3.11E-53	4.58E-34	y	n	n	n	-
				6.83	25.49	10	0	8	0							
ASA_P5G072	A4SUH4	AscX	Unknown function, chaperoned by AscY	5.51	4.90	5	2	4	1	8.63E-30	9.43E-13	y	n	n	n	-
				20.64	25.43	4	0	11	0							

The table shows fold changes for GP and SP in SNs (Log base 2 [wt/mut]) for PMSS and LFQ values, unique peptides, MS/MS Counts, PEP values, prediction for T3-effectors (Modlab and Effective) and secretion by alternative systems (SignalP and TatP), and the presence of eukaryotic domains in the protein.

AopH, Ati2 and AexT were the most secreted *A. salmonicida* proteins in wt SNs (GP or SP) (Figure 1, Additional files 1 and 2). When we calculated the ratio of [SN/pellet] quantities for each effector, we observed that AopP, AopH, AexT and Ati2 showed a high proportion in concentrated SNs, whereas this proportion was weak for AopO and AopN. This suggests that the in vitro secretion of AopO and AopN in wt SNs was significantly less efficient than AopP, AopH, AexT and Ati2.

We observed that AscX (ASA_P5G072) and ExsE (ASA_P5G062) were T3 secreted in wt SNs (Table 1). The same observation was made for YscX in *Yersinia pestis*[27]. YscX does not seem to be a T3SS effector, but it plays a role with its chaperone (YscY) and YscV in the export of needle components (YscF and YscI) [28]. In *Pseudomonas aeruginosa*, it was shown that the T3 secretion in extracellular medium and the T3 translocation into host cell of ExsE was required for transcriptional induction of the T3SS [29-31]. It is not known whether ExsE plays a role within the host cell.

Our proteomic analysis logically detected all translocon components (AopB, AopD, AcrV and AcrH) in *A. salmonicida* wt secretome (Figures 1 and 2, Table 1). AopB and AopD were among the top ten most abundant secreted proteins (Additional file 2). As expected, the elements of the T3SS needle (AscF, AscP, AscH and AscI) were also oversecreted in wt SNs and T3SS proteins of the OM ring (AscJ, AscD, AscC and ExsB), the inner membrane export apparatus (AscV, AscR, AscT and AscU) and the C ring/ATPase (AscL, AscK, AscN and AscQ) were only detected in pellets (Figure 2 and Additional files 1, 2 and 3).

Our study did not detect T3SS effectors AopX (homologuous to *V. parahaemolyticus* VopR [VP1683], *P. luminescens* plu4750) and ASA_0010 (homologuous to *V. parahaemolyticus* VopS [VP1686]) [32], suggesting that the mutations present in these genes in the genome of *A. salmonicida* A449 [12] and also in our wt strain prevent their production. However, the chaperone of VopS effector (ASA_0011) was detected, but only to a weak level in GP in the wt pellet.

From these results we concluded that our MS analysis localized 100% of T3SS components that are structurally linked to the bacteria and associated to pellets (cytoplasmic chaperones, OM, IM and C rings proteins) or T3 secreted and associated to SNs (effectors, translocon and needle elements) with effectiveness and accuracy. These results also support the idea that highly conserved cytoplasmic proteins unexpectedly present in *A. salmonicida* SNs and detailed in the first part of this work were not due to cell lysis.

The quantity of T3SS proteins was systematically lower in SP pellets (wt or mutant), and significantly lower in mutant pellets in comparison to wt (Additional files 1 and 3 for individual T3SS components), suggesting that the T3SS production was at a maximum when bacteria were in the phase of active multiplication and that the *ΔascV* knock-out mutation induced a strong down-regulation of the expression of many T3SS genes. AopD, AopB, AopH, AscV, Ati2, AcrV, AopO and AexT were the most abundant T3SS proteins present in the GP wt pellet (Additional file 3) and the difference in quantity (PMSS value) observed between the pellets of the wt and the *ΔascV* mutant in GP was confirmed by western blotting for AopD, AcrV and AexT (Additional file 4).

**Figure 3:**
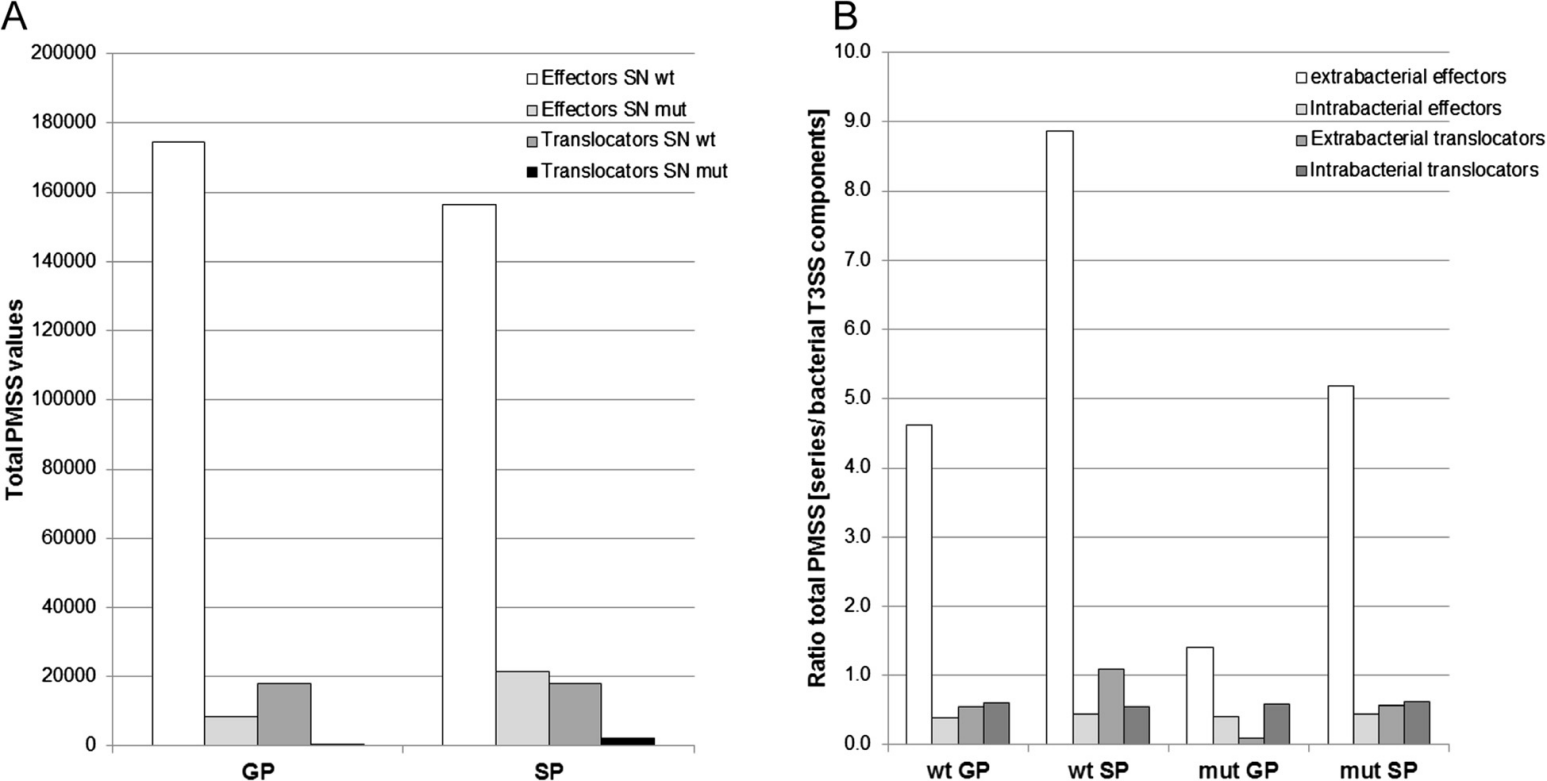
**Proportion of T3SS effectors and translocators.** The total PMSS value of T3SS effectors or translocators present in wt and mutant SNs during GP and SP are shown in diagram **(A)**. Quantities were strongly decreased in mutant SNs but low levels of T3SS effectors/translocators were detected and increased during SP. Diagram **(B)**, represents the proportion of [extra- or intrabacterial effectors or translocators/T3SS structural components] calculated with the total PMSS value of each category. These results showed that despite the *ascV* deletion, *A. salmonicida* kept the same proportion between intrabacterial effectors/translocators and the other components of the T3SS while strong differences were observed between wt and mutant for the proportion of extrabacterial effectors/translocators.

This underexpression of T3SS genes from different operons argues that the *ascV* deletion modulates the transcription regulation of several T3SS components and is not due to a polar effect. Strickingly, weak amounts of T3SS effectors/translocators were found in *ΔascV* mutant SNs (AopH, AexT, AopD, Ati2, AopP, AopN, AopB and ExsE by order of decreasing importance), but clearly to a lower extent than in wt SNs (Figure 3A and Additional files 1 and 2). As for the wt strain, the presence of these T3SS elements in mutant SNs was unlikely to be due to bacterial lysis given that (referred to the first part of this work for details): (i) ~90% of predicted cytoplasmic proteins in mutant pellets were never detected in SNs, (ii) GroEL, a marker of cell lysis, was among the most abundant proteins present in mutant pellets but was absent from SNs, and (iii) EF-Tu amount in mutant SNs decreased from GP to SP. The presence of T3SS effectors/translocators in mutant SNs was also unlikely to be due to a contamination between wt and mutant samples because, for example, the [wt/mutant] PMSS ratios of these T3SS-secreted components were 10-fold higher for AopP to 110-fold higher for AopB in GP SNs of wt when compared to *ΔascV* and were therefore not proportional. Burr and collaborators [2] did not detect AexT secretion in the *ΔascV* mutant SN, but they used unconcentrated SNs. Our samples were ~200 times more concentrated in this study. When we used total sum of PMSS values to calculate [intrabacterial effectors or translocators/T3SS structural components] we found that proportions were similar in wt and mutant strains (Figure 3B) assuming that, even if *ascV* was deleted, *A. salmonicida* kept the same proportion between the intracellular stock of effectors/translocators and the other T3SS structural components. As already mentioned, the proportion of [extrabacterial effectors or translocators/T3SS structural components] showed that the T3 secretion capacity was strongly impaired for the mutant strain during GP and SP, but this difference with the wt strain was weaker during the SP (Figures 3). This could mean that small amounts of effectors and translocators accumulated progressively in the mutant SNs along growth phases.

**Figure 4:**
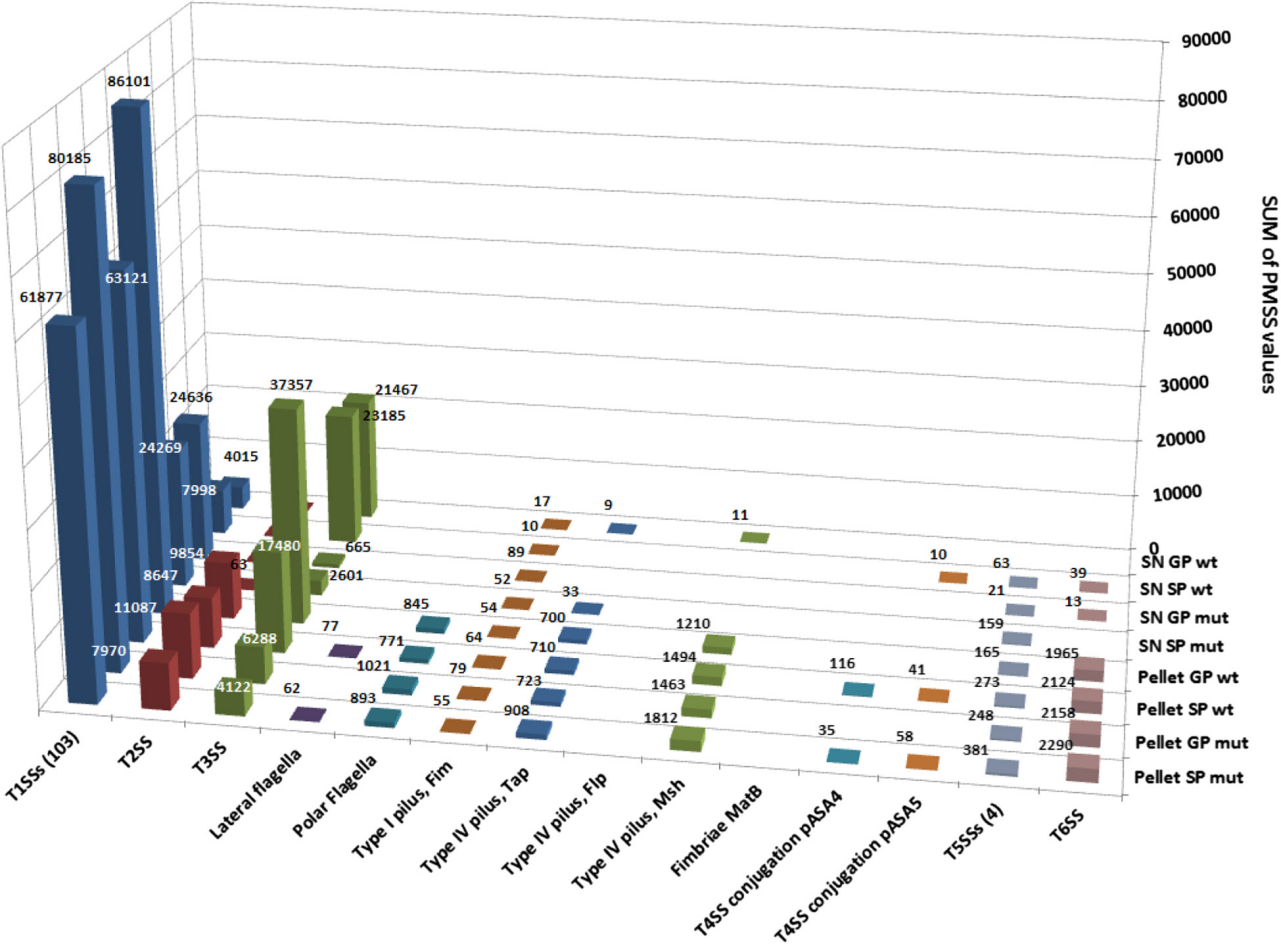
**Protein abundance of *A. salmonicida *appendages.** The diagram represents total PMSS values of *A. salmonicida* secretion systems (T1- to T6SS), flagella (lateral and polar), pili (T1 pilus [Fim], T4 pili [Tap, Flp and Msh] and fimbriae (MatB). Only the structural components were taken into account for the T3SS. Logically, the strongest difference in protein amount was observed for the T3SS. No differences were observed in pellets for T1- (103 ABC-transporters) and T2SS. In SNs, the strong difference between wt and mutant strains for T1SS values is due to the higher amount of VapA in mutant SNs.

The mutant strain might continue to release these T3SS components in SNs, either from the resting structural T3SS components or by an alternative secretion pathway. Recent publications argue that the T3SS arose from an exaptation of the flagellum, i.e. the recruitment of part of the flagellum structure for the evolution of the new protein delivery function [33,34] and, the secretion of T3SS effectors through flagella in the extracellular medium has been described in other bacteria [35,36]. The secretion of effectors/translocators by this process is unlikely in *A. salmonicida* given that functional lateral and polar flagella were not detected (Figure 4), thus confirming the results of studies showing that operons coding for *A. salmonicida* flagella contain several mutations [12]. However, we could imagine that FlhA (ASA_1351, polar flagella) and/or LfhA (ASA_0352, lateral flagella), showing respectively 56% and 55% of similarity with AscV might partially supply the function of this T3SS component. Such possible interactions between FlhA and the T3SS have been described in *Chlamydia pneumonia*[37]. While no mutations are predicted in these genes in *A. salmonicida* their expression was not detected in our pellets, but we cannot exclude that they were expressed below the detection limit of our system as our proteomic analysis did not cover the total proteome (59% of proteins common to all *Aeromonas* sp. were identified). Another possibility is that two mechanisms of effectors/translocators secretion operate in parallel along phases of growth, the first would be actively dependent on intact T3SSs while the second, clearly less efficient, would explain the progressive accumulation of effectors/translocators in the extracellular medium of the mutant strain. The alternative secretion of T3SS effectors through classical/unclassical pathways has never been described contrary to major constituents of the OM ring which are transported to the periplasm by the Sec-dependent secretion pathway [38]. Furthermore, the presence of T3SS effectors in the periplasma [39] and OMVs [40] has rarely been described. Another possibility might be the formation of double-bilayer OMVs (diameter from 100 to 250 nm) containing cytosolic components, as recently described in *Shewanella*[41], but GroEL would have been detected in SNs. Another study showed that in the absence of the host cell, at least YopH (homologous to AopH), YopE (AexT) and YopB/YopD translocators were excreted homogeneously at the *Yersinia* surface without physical association with the injectisome [42]. While the T3-dependent secretion of effectors in SNs is well-characterized, it is not known if these OM-associated effectors are excreted at the bacterial surface through a T3SS-dependent pathway. As a result, further investigations are necessary to clarify this point.

When we compared the total LFQ values of *A. salmonicida* secretion systems (T1- to T6SS), flagella (lateral and polar), pili (T1 pilus [Fim], T4 pili [Tap, Flp and Msh] and fimbriae (MatB), it was clear that the T3SS was the most expressed system by *A. salmonicida* (Figure 4). T1- and T2SS were expressed just as much in wt and mutant pellets, showing that their expression and function was not impaired by the knock-out mutation in *ascV*. All of the other systems (flagella, pili, fimbriae, T4-, T5- and T6SS) were either not expressed at all or were expressed to a lower level, suggesting that they could be impaired by mutations similar to the ones observed in the reference A449 strain [12].

### Other putative virulence factors oversecreted in *A. salmonicida* wt SNs

We combined several thresholds to identify additional putative *A. salmonicida* T3SS effectors and T3-independent virulence factors. We targeted wt secreted proteins with PMSS values over 25, a PMSS or LFQ intensity 4-fold increased in the wt SN, and a PEP value inferior to 10^-8^ or equal to zero (Figure 2 and Table 2). We then performed bioinformatics analyses to predict whether a peptide signal for Sec-, Tat- or T3 dependent secretion was present in the N-terminal part of secreted proteins.

**Table 2:** **Oversecreted proteins by *A. salmonicida *wt strain**

**Locus**	**Uniprot**	**Name**	**Description**	**Fold change in SNs Log**_**2 **_**(wt/mut) [PMSS]**^**1 **^**and [LFQ]**^**2**^		**[Unique peptides]**^**3 **^**and [MS/MS Count]**^**4**^				**PEP GP**	**PEP SP**	**Modlab (>0.4)**	**Effective (>0.95)**	**SignalP**	**TatP**	**Eukaryotic domain**
				**GP**	**SP**	**wt GP**	**mut GP**	**wt SP**	**mut SP**							
**With a predicted signal for T3SS**																
ASA_0292	A4SHV8	EF-G	Elongation factor G	2.42^**1**^	0.98	33^**3**^	25	41	40	0.00E + 00	0.00E + 00	y	n	n	n	-
				3.60^**2**^	1.27	73^**4**^	19	181	106							
ASA_0275	A4SHU2	EF-Tu	Elongation factor Tu	1.03	1.08	24	21	24	23	2.98E-220	0.00E + 00	n	y	n	n	PF03143
ASA_0293				2.49	2.15	52	34	118	81							PF01926
ASA_2996	A4SQ25	DnaK	Chaperone protein DnaK	2.91	0.85	30	22	37	37	2.28E-272	0.00E + 00	y	n	n	n	PF00012
				3.27	0.30	59	11	150	78							
ASA_1014	A4SJR9	Pnp	Polyribonucleotide nucleotidyltransferase	7.94	1.33	15	14	17	16	1.55E-89	6.02E-126	y	y	n	n	PF03726
				3.42	0.52	23	1	36	17							PF13014
																PF07650
ASA_1826	A4SLY0	HtpG	Chaperone protein HtpG	2.80	7.78	18	14	24	21	7.44E-102	2.58E-181	n	y	n	n	-
				5.40	4.83	27	3	44	1							
ASA_2347	A4SNC4	MdeA	Methionine gamma-lyase	1.98	0.35	10	8	8	8	1.14E-68	2.52E-51	n	y	n	n	PF00155
				1.45	0.80	15	5	26	24							PF00266
ASA_1990	A4SMD9	PepN	Aminopeptidase N	5.81	4.67	5	4	3	4	1.14E-14	1.00E-13	n	y	n	n	PF11940
				4.97	4.37	6	0	6	1							PF01433
ASA_4301	G7D0H6	OpdA	Oligopeptidase A	4.21	0.00	3	3	2	4	1.61E-08	2.78E-08	y	y	n	n	PF01432
				3.33	2.12	3	0	3	0							
**Without any signal for secretion but having homologues T3 secreted in other bacteria**																
ASA_P5G088	A4SUI8	-	α/β hydrolase	2.50	1.81	9	6	5	6	2.08E-56	8.03E-19	n (0.36)	n	n	n	PF12697
				2.16	-2.05	12	3	12	10							PF12695
																PF02129
																PF00561
																PF00326
																PF12146
																PF01738
																PF08840 PF07859
ASA_3402	A4SR55	Pta	Phosphate acetyltransferase	7.13	1.42	9	7	4	3	2.98E-87	5.28E-66	n	n	n	n	PF01515
				3.55	-0.39	8	0	8	2							
**Without any signal for secretion**																
ASA_4119	A4ST37	TypA (BipA)	GTP binding protein	2.12	2.24	14	12	19	18	2.93E-98	1.52E-178	n	n	n	n	PF01926 PF08477
				2.69	1.84	31	11	49	12							
ASA_1768	A4SLS5	RpsA	30S ribosomal protein S1	2.62	1.77	15	13	7	7	1.27E-135	1.40E-78	n	n	n	n	-
				7.33	3.00	14	1	9	4							
ASA_1202	A4SK88	TktA	Transketolase 1	0.88	2.43	13	12	20	19	1.34E-82	8.67E-175	n	n	n	n	PF00456
				2.67	1.22	14	13	32	8							PF13292
ASA_0427	A4SI83	AcnB	Aconitate hydratase 2	6.86	0.78	13	6	11	11	3.46E-52	2.17E-117	n	n	n	n	PF00330
				5.65	1.77	13	0	20	10							
ASA_4076	A4SSZ5	RplX	50S ribosomal protein L24	6.01	1.86	5	4	9	9	4.94E-38	4.46E-89	n	n	n	n	PF00467
				1.11	1.00	5	0	37	23							
ASA_4087	A4ST06	RplC	50S ribosomal protein L3	2.24	5.67	6	5	6	6	1.14E-65	5.13E-33	n	n	n	n	PF00297
				3.03	2.24	26	12	16	4							
ASA_0684	A4SIX5	IleS	Isoleucine-tRNA ligase	5.92	2.17	7	7	13	13	4.17E-61	2.45E-50	n	n	n	n	PF00133
				3.14	0.39	11	0	18	10							PF08264
																PF09334
																PF13603
																PF01406
																PF06827
ASA_1068	A4SJW8	LeuS	Leucine tRNA ligase	4.86	1.11	3	3	2	3	7.59E-21	1.04E-18	n	0.91	n	n	PF13603
				3.17	4.42	2	0	2	0							PF00133
																PF09334
																PF08264
ASA_0707	A4SIZ7	RpsF	30S ribosomal protein S6	5.15	5.74	3	3	4	3	2.90E-24	1.66E-10	n	n	n	n	-
				-14.13	7.23	2	0	19	2							
ASA_1442	A4SKW9	WecB	UDP-N-acetylglucosamine 2-epimerase	1.65	0.99	4	3	6	6	1.41E-30	9.18E-24	n	n	n	n	PF02350
				21.57	1.21	2	0	7	5							
**With a signal for secretion by the Sec-dependent pathway**																
ASA_1267	A4SKF2	OmpAI	Outer membrane protein AI	7.22	6.94	8	5	11	6	9.60E-91	1.27E-145	n	n	y	n	-
				1.02	3.43	12	0	37	0							
ASA_1544	A4SL60	OmpK40	Outer membrane protein K40	5.80	5.70	8	8	7	6	4.58E-23	7.69E-85	n	n	y	n	-
				2.32	6.93	10	1	16	0							
ASA_3619	A4SRQ8	-	Hypothetical ABC-type Fe3 + -hydroxamate transport system component	5.27	4.08	3	2	6	5	8.50E-36	4.00E-16	n	n	y	n	-
				4.71	5.77	5	0	21	3							
ASA_0330	A4SHZ1	DegQ	Serine protease	1.97	0.00	5	5	2	2	3.03E-43	4.23E-69	n	n	y	n	PF13180
																PF00089
				3.24	2.59	7	5	3	0							
ASA_0716	A4SJ06	-	Insulinase, peptidase M16	2.61	0.53	16	11	11	11	6.18E-98	6.71E-125	n	n	y	n	PF05193
				4.55	1.28	22	3	12	9							PF00675
ASA_0852	A4SJC4	-	Hypothetical outer membrane lipoprotein	4.92	6.39	4	1	3	3	9.71E-08	5.69E-56	n	n	y	n	-
				20.61	4.48	7	0	10	0							

The table shows fold changes for GP and SP in SNs (Log base 2 [wt/mut]) for PMSS and LFQ values, unique peptides, MS/MS Counts, PEP values, prediction for T3-effectors (Modlab and Effective) and secretion by alternative systems (SignalP and TatP), and the presence of eukaryotic domains in the protein.

From 466 proteins detected in SNs, only 26 proteins were more abundant in wt than in mutant SNs, while their presence was approximatively similar in pellets. Among the first targeted proteins, seven were surprisingly designated by bioinformatics as T3 effectors (EF-G, EF-Tu, DnaK, HtpG, PNPase, MdeA, PepN and OpdA), and two proteins without a predicted motif for T3 secretion were shown to have homologues that are T3 secreted in other bacteria (Pta and ASA_P5G088) (Table 2). These proteins were secreted to a clear lesser extent than previously described T3SS effectors, and these results should therefore be interpreted with caution and need further investigations in order to confirm that they are (T3-) secreted. Strikingly, homologues of these proteins are present in eukaryotic cells, where they play fundamental roles and sometimes alternative (moonlighting) functions (EF-1*α* for EF-Tu [43], HSP70 and HSP90 for DnaK and HtpG [44-46], eukaryotic aminopeptidases and thimet oligopeptidase for PepN and OpdA [47-49]). For example, these molecular chaperones play a role in the virulence of other pathogens and are considered as new targets for therapy [50,51]. It is tempting to assume that EF-G, EF-Tu, DnaK, HtpG, PepN and OpdA might be injected by *A. salmonicida* into host cells in order to interfere with these functions.

Polynucleotide phosphorylase PNPase has pleiotropic roles in bacteria such as degrading mRNA (degradosome) and mediating post-transcriptional regulation [52]. However, it was shown that PNPase was required for the optimal functioning of *Yersinia* T3SS and enhanced the ability of the bacterium to withstand the killing activities of murine macrophages [53]. In *Salmonella enterica* and *Dickeya dadantii*, PNPase downregulated the transcription of T3SS genes [54,55].

Although they did not have the N-terminal motif for T3-secretion, the phosphate acetyl transferase (Pta, ASA_3402) and the putative α/β hydrolase ASA_P5G088 of *A. salmonicida* were targeted by our screening as putative T3SS effectors. In *Salmonella*, a Pta mutant showed that this enzyme was associated to virulence [56], and a recent study demonstrated that *E. coli* Pta (E2348C_2437, 83% similarity with *A. salmonicida* Pta) might be secreted by the T3SS [57]. Interestingly, homologues of ASA_P5G088 in *V. parahaemolyticus* (35% and 38% of similarity with VP1677 and VP1678) were T3 secreted [58].

Ten cytoplasmic proteins were more abundant in wt vs *ΔascV* mutant SNs, did not have any predicted signal for a secretion system and were not characterized as T3SS effectors in other bacteria (Table 2). TypA (or BipA) is a GTPase that was associated to virulence [59,60] through regulation of the T3SS [61,62]. Interestingly, even though the TypA N-terminal part does not contain a predicted signal for T3 secretion, it shares three conserved motifs with the N-terminal part of EF-G and EF-Tu. Unclearly, ribosomal protein 30S S1, 30S S6, 50S L24 and L3, IleS, LeuS, Tkt, AcnB, and WecB were more abundant in wt SNs. All of these components were discovered to be associated to the *A. salmonicida* surfacome and in the secretome of other bacteria (refer to the Additional file 8 of the Part 1 of this work, “*The Aeromonas salmonicida subsp. salmonicida exoproteome: global analysis, moonlighting proteins and putative antigens for vaccination against furunculosis*” for details). AcnB and WecB have homologous proteins that have been associated to the virulence in other bacteria (Additional file 5).

Six proteins with a predicted T1 peptide signal were systematically found either in higher amounts or only in wt SNs compared to the *ΔascV* mutant (Table 2). That was the case for OmpAI (ASA_1267) and OmpK40 (ASA_1544), which were linked to virulence in *Aeromonas* and other bacteria (Additional file 5). The presence of these OM proteins in SNs was not an artefact given that OmpAII (ASA_1266) was produced just as much in pellets as OmpAI but was never detected in SNs (Additional file 1). The periplasmic trypsin-like serine protease DegQ (ASA_0330), the insulinase ASA_0716 (zinc-dependent peptidase M16), the putative OM lipoprotein ASA_0852, and the putative ABC-type Fe3 + -hydroxamate transport system component ASA_3619 were also increased in wt SNs, and such proteins have also been related to virulence in other bacteria (Additional file 5). Interestingly, *A. hydrophila* homologues of ASA_0852 and ASA_3619 were found in all toxic extracellular product fractions of the bacterium [63].

### Analysis of previously-described and newly detected putative virulence factors

**Figure 5:**
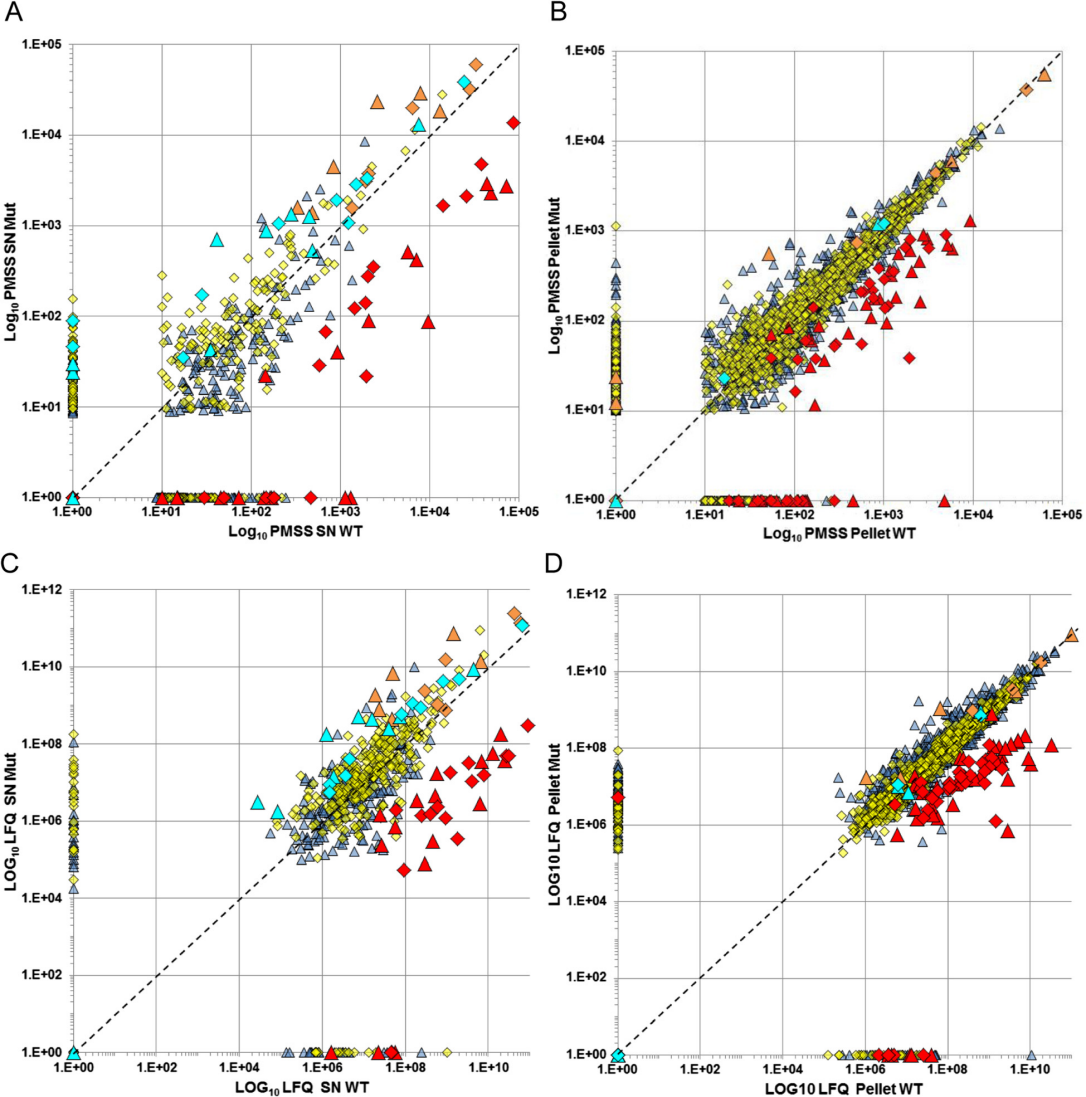
**Correlation of protein contents between wt and T3SS-mutant.** Each plot represents the PMSS **(A and B)** or LFQ **(C and D)** values for each protein identified in wt (X-axis) and/or mutant (Y-axis) strains, in supernatants (SNs, **A** and **C**) and pellets **(B and D)**. Values of exponential growth phase (GP) are dark blue triangles and stationary phase (SP) values are yellow squares. The global distribution of wt vs mutant protein values was linear in all conditions, but with a larger repartition in SNs than in pellets, thereby indicating differences in protein secretion between wt and *ΔascV* strains. Red values = T3SS proteins; orange = VapA, AerA, AerB, Ahe2, SatA and Asx; light blue = TagA, microbial collagenase, extracellular desoxyribonuclease ASA_1199, PlaA1, PlaC, Amy1, CdxA, ChiB, Chi2 and AmyA.

Besides the T3SS, other virulence factors of *A. salmonicida* have been characterized (or predicted) in the literature, and certain conserved proteins are homologous to virulent toxins, adhesins and enzymes identified in other bacteria (Table 3, Additional file 5). We identified the tetragonal surface virulence array protein VapA, aerolysin AerA, hemolysin AerB, esterase SatA, extracellular phospholipase PlaA1, phospholipase PlaC, the metalloprotease/mucinase, serine protease Ahe2, chitin/ N-acetylglucosamine-binding protein (ASA_0604), extracellular nuclease (ASA_1199), enolase (ASA_3475), and outer membrane endopeptidase PepO. Our results showed that all these toxins and enzymes were secreted as much as or more as in the extremely low-virulent *ΔascV* mutant (Figure 5, Table 3 and Additional file 2) and they highlighted that an intact T3SS is primordial to initiate the disease. This observation is supported by studies demonstrating that the deletion of T3SS genes completely abolishes the virulence [2,3,13-16,24,64].

**Table 3:** **Other characterized or putative virulence factors of *A. salmonicida***

**Locus**	**Uniprot**	**Name**	**Description**	**Fold change in SNs wt/mut [PMSS]**^**1 **^**and [LFQ]**^**2**^		**[Unique peptides]**^**3 **^**and [MS/MS Count]**^**4**^				**PEP GP**	**PEP SP**	**Modlab (>0.4)**	**Effective (>0.95)**	**SignalP**	**TatP**	**Eukaryotic domain**
				**Log**_**2 **_**(wt/mut) GP**	**Log**_**2 **_**(wt/mut) SP**	**wt GP**	**mut GP**	**wt SP**	**mut SP**							
**Other characterized virulence factors**																
ASA_1438	A4SKW5	VapA	Tetragonal surface virulence array protein	-3.18^**1**^	-1.66	35^**3**^	42	39	40	0.00E + 00	0.00E + 00	n	n	y	n	-
				-7.09^**2**^	-4.02	284^**4**^	2096	1203	3044							
ASA_3906	A4SSI7	AerA	Aerolysin A	-2.43	-0.69	18	28	27	28	7.56E-292	0.00E + 00	n	n	y	n	PF03318
				-6.58	-0.85	79	351	358	560							
ASA_2854	A4SPP5	AerB	Aerolysin B	-2.28	-0.20	24	25	33	33	7.45E-255	0.00E + 00	n	n	y	n	PF00652
				-3.20	0.28	25	161	259	284							
ASA_0509	A4SIF4	SatA	Glycero-phospholipid-cholesterol acyltransferase	-1.54	-0.89	16	15	24	24	2.62E-221	0.00E + 00	n	n	y	n	
				-5.08	-2.95	42	110	393	669							PF00657
																PF13472
ASA_4288	A4STJ0	PlaA1	Phospholipase A1	-0.14	0.21	22	23	24	23	0.00E + 00	0.00E + 00	n	n	n	n	-
				-2.66	-1.87	31	39	121	119							
ASA_0635	A4SIS6	PlaC	Phospholipase C	-4.90	-6.49	2	5	8	10	2.22E-14	4.03E-81	n	n	y	n	PF03372
				-6.86	-2.01	0	5	4	20							
ASA_3321	A4SQY1	TagA	Metalloprotease/mucinase ToxR-regulated lipoprotein	-2.28	-0.92	30	31	38	38	5.47E-206	0.00E + 00	n	n	y	n	-
				-6.12	-2.38	30	137	230	406							
ASA_2540	A4SNU7	Ahe2	Serine protease	-1.91	-0.86	31	34	49	49	0.00E + 00	0.00E + 00	n	n	y	n	PF01483
				-5.65	-2.51	529	1363	4391	6633							
ASA_0604	A4SIP8	ChiY	Chitin-binding protein	-3.24	-1.03	15	19	22	24	2.64E-275	0.00E + 00	n	n	n	n	PF03067
				-6.29	-1.94	17	124	309	548							
ASA_1199	A4SK85	Nuc	Extracellular desoxyribonuclease	NV	-2.61	0	0	9	9	NV	4.65E-86	n	n	y	n	PF04231
					-3.19	0	0	6	20							
ASA_3475	A4SRC1	Eno	Enolase	0.85	-0.98	15	17	17	20	6.58E-271	0.00E + 00	n	n	n	n	PF00113
																PF03952
				0.44	-1.70	50	35	34	80							
																PF07476
ASA_3132	A4SQF4	PepO	Peptidase M13	NV	NV	NV	NV	NV	NV	NV	NV	n	n	y	n	PF05649
																PF01431
**Putative virulence factors**																
ASA_0826	A4SJA3	Asx	RTX large exoprotein	-0.50	-0.22	92	90	106	107	0.00E + 00	0.00E + 00	n	n	n	n	PF00092
				-1.11	-1.15	1372	1991	3830	4248							
ASA_3723	A4SS12	-	Microbial collagenase	-0.83	-0.67	66	64	80	80	0.00E + 00	0.00E + 00	n	n	y	n	-
				-0.96	-0.90	529	974	3304	4967							
ASA_2541	A4SNU8	-	Unknown	-2.20	-1.01	11	11	13	14	1.91E-144	0.00E + 00	n	n	y	n	-
				-5.88	-3.83	121	582	1571	2701							
ASA_2206	A4SMZ6	NucH	Nuclease	-2.09	-0.69	39	43	41	41	0.00E + 00	0.00E + 00	n	n	y	n	PF03160
				-5.38	-1.48	75	289	215	345							PF03372
ASA_3073	A4SQ99	-	Leucine aminopeptidase	-2.31	-0.78	13	14	17	17	1.39E-191	0.00E + 00	n	n	y	n	PF04389
				-5.13	-2.04	40	157	1074	1519							PF01546
pRA1_0073	C6GA30	-	Group 3 Ig-like domain protein	-0.80	-0.34	43	42	85	85	0.00E + 00	0.00E + 00	n	n	y	n	-
				-1.30	-1.33	67	108	859	992							
ASA_P4G163	A4SU89	-	Group 3 Ig-like domain	-0.59	-0.36	10	10	16	16	1.01E-102	0.00E + 00	n	n	y	n	-
				-1.65	-1.21	19	36	171	207							
ASA_0873	A4SJD6	CdxA	Chitinase	-1.50	-0.76	27	29	50	51	0.00E + 00	0.00E + 00	n	n	y	y	PF00704
				-2.75	-1.31	48	165	305	553							PF02839
ASA_2142	A4SMT5	Chi2	Chitinase	-4.10	-2.39	11	17	22	25	5.49E-126	0.00E + 00	n	n	y	n	PF00182
				-7.15	-2.91	5	86	56	235							PF02839
ASA_3320	A4SQY0	ChiB	Chitinase	-2.59	-1.07	13	13	27	27	4.47E-159	0.00E + 00	n	n	y	n	PF00182
				-4.79	-2.82	19	94	180	370							PF02839
ASA_3982	A4SSR0	Taxi	TRAP-associated extracytoplasmic immunogenic	0.51	-1.50	11	9	16	18	4.95E-131	0.00E + 00	n	n	y	n	PF09084
				0.22	0.20	18	18	63	127							
ASA_0849	G7CXH6	PrtV	immune inhibitor A metalloprotease	-0.16	1.19	16	17	29	29	1.92E-129	0.00E + 00	y	n	n	n	-
				-2.54	0.67	17	28	169	82							
ASA_1287	A4SKH1	LasA	Metalloprotease	-2.82	-1.45	10	13	13	13	2.90E-123	0.00E + 00	n	y	n	n	PF01551
				-6.19	-4.03	16	81	146	349							
ASA_1027	A4SJT2	-	LysM domain-containing protein	-0.57	1.00	10	10	12	12	1.92E-87	4.24E-187	n	n	y	n	PF01476
				-1.71	0.46	20	32	135	87							
ASA_1998	A4SME7	-	GlyGly-CTERM protein	-1.80	-1.20	21	24	29	29	3.33E-303	0.00E + 00	n	n	y	n	-
				-2.17	-1.01	26	91	115	226							
ASA_1199	A4SK85	-	Extracellular desoxyribonuclease	NV	-2.61	NV	NV	9	9	NV	4.65E-86	n	n	y	n	PF04231
					-3.19			6	20							
ASA_P4G031	A4STW2	-	Micrococcal nuclease (SNase-like)	-0.45	-5.77	3	3	6	7	1.24E-04	9.28E-20	n	y	n	n	PF00565
				-0.51	-3.82	3	4	3	29							

The table shows fold changes for GP and SP in SNs (Log base 2 [wt/mut]) for PMSS and LFQ values, unique peptides, MS/MS Counts, PEP values, prediction for T3-effectors (Modlab and Effective) and secretion by alternative systems (SignalP and TatP), and the presence of eukaryotic domains in the protein.

NV: No value, not detected.

Our proteomic study also characterized, the secretion in SNs of other putative virulent toxins, adhesins and enzymes conserved among *Aeromonas* sp. for the first time (Table 3, Additional file 5), in decreasing order of quantity in SNs (Additional file 2): the large RTX (repeats in toxin) exoprotein Asx (ASA_0826), a microbial collagenase (ASA_3723), an unknown protein ASA_2541 that could be co-expressed/secreted with Ahe2, the nuclease NucH (ASA_2206), a leucine aminopeptidase (ASA_3073), two large unknown proteins with a Ig-like domain (homologues to pRA1_0073 in IncA/C plasmids and ASA_P4G163), chitinases CdxA, Chi2 (ASA_2142) and ChiB (ASA_3320), the solute receptor TAXI (TRAP-associated extracytoplasmic immunogenic) of a TRAP transporter (ASA_3982), the immune inhibitor A metalloprotease PrtV (ASA_0849), the metalloprotease LasA, a LysM domain-containing protein (ASA_1027), the hypothetical GlyGly-CTERM protein ASA_1998, the micrococcal nuclease (SNase-like) ASA_P4G031, the azurin, and the Type I pilus subunit FimD. All of these proteins were as much as or more secreted in mutant SNs (Table 3 and Additional file 2), highlighting once again that an intact T3SS is primordial to initiate the disease. The putative hemolysin ASA_1523 was only detected in pellets and in higher quantity in the mutant strain.

In the genome of *A. salmonicida* A449, Zonular Occludens Toxins (Zot, ASA_2003 and ASA_2015), elastase AhpB and toxic extracellular endopeptidase AsaP1 genes are impaired by deletions and insertion elements. According to these observations, we did not detect any polypeptides for these proteins in our MS experiments, suggesting that they would be also disrupted in our *A. salmonicida* strain. Furthermore, the insecticidal cytolytic delta-endotoxin (ASA_2128), putative RTX toxins (ASA_0127, ASA_1674 and ASA_1675), a secreted metalloprotease (ASA_1723) and the pullulanase PulA were not identified, and their expression might be induced in the host. Finally, 15 prophage proteins were identified in pellets (12% of prophage genes detected in the genome of the reference strain A449) and only one (ASA_2013) was detected in SNs, but without any significant differences between the wt and mutant strains.

## Conclusions

The comparison by high-throughput proteomics of *A. salmonicida* secretomes from wt and T3SS-deficient strains is a powerful method that gave us the opportunity (i) to characterize the full in vitro repertoire of T3SS effectors represented mainly by AopH, Ati2, AexT, AopP, AopO, AopN and ExsE, (ii) to identify new putative virulence factors that are secreted in the extracellular medium or might be translocated into the host cell by the T3SS or alternative mechanisms, and (iii) to confirm that *A. salmonicida* secreted toxins, adhesins and enzymes that have been described until now and are additionally found in this study are secreted to a higher extent in the extremely low-virulent *ΔascV* mutant.

Our results also clearly show that the deletion of one gene (*ascV* in this study) can induce the down-regulation of several other genes (only associated to the T3SS in our study), not necessary transcriptionally linked in the same operon. To respect the molecular Koch’s postulates, we can conclude from this study that each work investigating phenotypic characters by site-directed mutagenesis should ideally be completed by a larger analysis studying the impact of the mutation over the global gene expression.

Due to the fact that we studied in vitro secretomes, T3SS effectors that we have found might be considered as the first line of weapons that *A. salmonicida* uses to invade fish and initiate the disease. Inside the salmonid, bacteria might induce the expression of genes specific to the *A. salmonicida* species and present in genomic islands (such as the cluster of genes [ASA_1049 to ASA_1052] homologous to the Vibrio Seventh pandemic Island-I [VSP-I]) that might be necessary to survive in new environments [65]. Interestingly, T3SS effectors predicted by bioinformatics are two times more abundant in genomic regions specific to *A. salmonicida* (15% of specific genes) than in genetic regions common to all *Aeromonas* species (8% of common genes). Further proteomics studies will be necessary in order to confirm the in vivo *A. salmonicida* secretome.

## Methods

### Cell culture and preparation of bacterial supernatants and pellets for LC-MS/MS

*Aeromonas salmonicida* wt and *ΔascV* mutant strains used in this study were characterized in a previous work [15]. To get *A. salmonicida* wt cultures into a maximum T3SS activation state, we used JF2267 strain which was freshly reisolated from an experimentally infected dead fish (JF5054). This re-isolated strain was highly virulent, since intraperitoneal inoculation of only 500 cfu per fish was sufficient to induce 70 to 80% of mortality in challenge assays [22]. The *ΔascV* mutant strain JF2747 is considered to have extremely low-virulence because 10^5^ cfu/fish induced no mortality [15], and 10^8^ cfu/fish induced a weak mortality of only 20%.

To precipitate and concentrate proteins from the supernatant of wt and *ΔascV A. salmonicida*, 50 ml of TSB medium were inoculated with 10^9^ bacteria and cultivated at 18°C under shaking (160 rpm) in the presence of protease inhibitors (Complete, Roche Diagnostics). The bacterial growth was stopped during the exponential phase of growth (DO_600_ = ~1.5) and the stationary phase (DO_600_ >2.0). Supernatants were separated from bacterial pellets by centrifugation (6.000 × g, 10 min, 4°C) and filtration through a 0.22 μM Acrodisc filter (low protein binding, PALL Life Sciences). The bacterial pellets were resuspended in 10 ml of PBS, and 250 μL of these solutions were mixed with 250 μL of SDS loading buffer and heated at 100°C for 5 min. To precipitate proteins from supernatants, 12.5 ml of 100% ice-cold trichloroacetic acid were added to the solutions (20% final concentration), then immediately vortexed and incubated overnight on ice. Supernatants were removed and brown protein pellets were suspended and washed several times by centrifugation in ice-cold 100% acetone in 2 ml Eppendorf tubes (low binding protein). Finally, the pellets were dried, diluted in 250 μL of SDS loading buffer (~200 times concentration) and heated at 100°C for 5 min. Proteins were separated in non-adjacent wells (to avoid well to well contamination) on 15% acrylamide SDS-PAGE gels and stained with Coomassie. One run for each of the eight biological conditions (wt vs mutant, GP vs SP and SN vs pellet) was completely sliced from the stacking gel to the buffer front in 20 to 25 bands, and each band was cut into small (~1 mm^3^) cubes for protein in-gel digestion and MS analysis, as described elsewhere [66,67]. Peptide sequencing was made on a LTQ Orbitrap XL mass spectrometer (ThermoFisher Scientific, Bremen; Germany) equipped with a Rheos Allegro nano flow system with AFM flow splitting (Flux Instruments, Reinach; Switzerland) and a nano electrospray ion source operated at a voltage of 1.7 kV. Peptide separation was performed on a Magic C18 column (5 μm, 100 Å, 0.075 × 70 mm) using a flow rate of ~400 nL/min and a linear gradient of 5 to 40% acetonitrile in water/0.1% (v/v) formic acid during 60 min.

The mass spectrometry proteomics data were deposited to the ProteomeXchange Consortium (http://proteomecentral.proteomexchange.org) via the PRIDE partner repository [68] with the dataset identifier PXD000429 and DOI 10.6019/PXD000429.

### LC-MS/MS data interpretation

LC-MS/MS data interpretation was made against the current UniProtKB database release (2012_06) of all known *A. salmonicida* protein sequences. Two methods of relative protein quantification were used. The peptide-matching score summation (PMSS) is a label-free technique that assumes ideal scoring for proteins as the summative of the identification scores of their constituent peptides freed upon digestion. A higher score represents a more abundant protein [69]. The EasyProt search algorithm [70] was used for this, as described in [67]. The obtained mass spectrometric raw data were also analyzed with MaxQuant, version 1.2.2.5 [71], and its label-free quantitation (LFQ) algorithms [72] allowed quantitative comparisons. MaxQuant settings were as follows: Accepted false discovery rates at peptide, modified peptide and protein level were set at 1% using the reversed sequence database. Carbamidomethylation on Cys was set as a fixed modification. Oxidation of Met, acetylation on protein N-terminus, and phosphorylation on Set/Thr/Tyr were set as variable modifications with a precursor mass tolerance of 6 ppm in the main search, while only oxidation and acetylation with a mass accuracy of 20 ppm was used in the first search. Trypsin cleavage specificity was set at full with a maximum 2 missed cleavages and the allowance of up to three modifications per peptide of length between 6–25 amino acids. Fragment spectra were filtered to the 6 most intense peaks per 100 Da mass windows and searched with a mass tolerance of 0.5 Da. Protein identifications were accepted with at least 2 razor and unique peptide identifications. For label free quantification (LFQ), at least 2 unmodified or acetylated protein N-terminal peptides were required, and matching within a 2 minute time frame between samples was allowed. Only proteins with significant increased PMSS and LFQ values in GP and SP of wt vs mutant SNs were developed in the text.

### Bioinformatics analysis

Detection of signal sequences for secretion was carried out using the SignalP 4.1 server (http://www.cbs.dtu.dk/services/SignalP/) [73], TatP 1.0 server (http://www.cbs.dtu.dk/services/TatP/) [74] and the T3SS effector prediction softwares from Modlab® (http://gecco.org.chemie.uni-frankfurt.de/T3SS_prediction/T3SS_prediction.html) [75] and EffectiveT3 (http://www.effectors.org/) [76]. The list of *A. salmonicida* ABC transporters was provided by ABCdb (https://www-abcdb.biotoul.fr/) [77] and prohage genes by PHAST (http://phast.wishartlab.com/index.html) [78].

## Abbreviations

APC: Antigen presenting cell; CTL: Cytotoxic T lymphocytes; GP: Exponential phase of growth; LFQ: Label-free quantitation; PMSS: Peptide-matching score summation; SN: Supernatant; SP: Stationary phase of growth; T3SS: Type-three secretion system; wt: wild-type.

## Competing interests

The authors have declared that no competing interests exist.

## Authors’ contributions

PVB conceived of the study, carried out the experiments, analyzed data from MS, performed bioinformatic analyses and drafted the manuscript. SB-L and MH performed MS experiments and interpretation of MS data. JF helped to draft the manuscript. Authors read and approved the final manuscript.

## Supplementary Material

Additional file 1 **Table: PMSS, LFQ values, ratios, PEP values, subcellular localization, secretion system signals for each protein identified in SNs and pellets of wt and mutant strains in GP and SP.** Column B: Proteins names. Red = T3SS components; dark red = other virulence factors (toxins, enzymes and adhesins); light red = putative secondary virulence factors; yellow = proteins specific of JF2267 or B526; mauve = multidrud resistance-associated proteins; orange = ABC transporters; light green = proteins associated to flagella, pili, T4SS; dark blue = phage proteins; light blue = cytoplasmic moonlighting proteins present in SNs; grey = T5SS; light pink T6SS, pink: transposases. Column E: A449 Loci. Grey = genes conserved among *Aeromonas* sp.; white = genes shared with at least one other *Aeromonas* species; green = genes specific of *A. salmonicida*; yellow = genes specific of *A. salmonicida* JF2267 and B526; pink = transposases. Click here for file

Additional file 2 **Figure: most abundant proteins in *A. salmonicida* supernatants of wt and mutant strains in GP and SP.** The diagram represents the most abundant proteins secreted by *A. salmonicida* (in decreasing order of PMSS values in wt SN during GP). Below the name of the protein circles represent T3SS components (red), other virulence factors (toxins, enzymes and adhesins) (pink), putative secondary virulence factors (yellow) and cytoplasmic proteins with putative moonlighting activity (green). Click here for file

Additional file 3 **Figure: most abundant proteins in *A. salmonicida* pellets of wt and mutant strains in GP and SP.** The diagram represents the most abundant proteins detected in *A. salmonicida* pellets (in decreasing order of PMSS values in wt pellet during GP). Below the name of the protein circles represent T3SS components (red), other virulence factors (toxins, enzymes and adhesins) (pink), putative secondary virulence factors (yellow) and secreted cytoplasmic proteins with putative moonlighting activity (green). Click here for file

Additional file 4 **Figure: confirmation by western blotting of the difference in quantity observed between the pellets of the wt and the *ΔascV *mutant in the GP for AopD, AcrV and AexT.** Click here for file

Additional file 5 **Table: *A. salmonicida* secreted proteins that have homologues in other bacteria with a putative role in virulence.** Click here for file
