# Supplementary figures and images for: The Aeromonas salmonicida subsp. salmonicida exoproteome: determination of the complete repertoire of Type-Three Secretion System effectors and identification of other virulence factors
Source: Proteome Sci. 2013 Sep 27;11:42. doi: 10.1186/1477-5956-11-42 (PMC3852671; doi:10.1186/1477-5956-11-42)

PMSS values of wt and mutant SNs in GP and SP

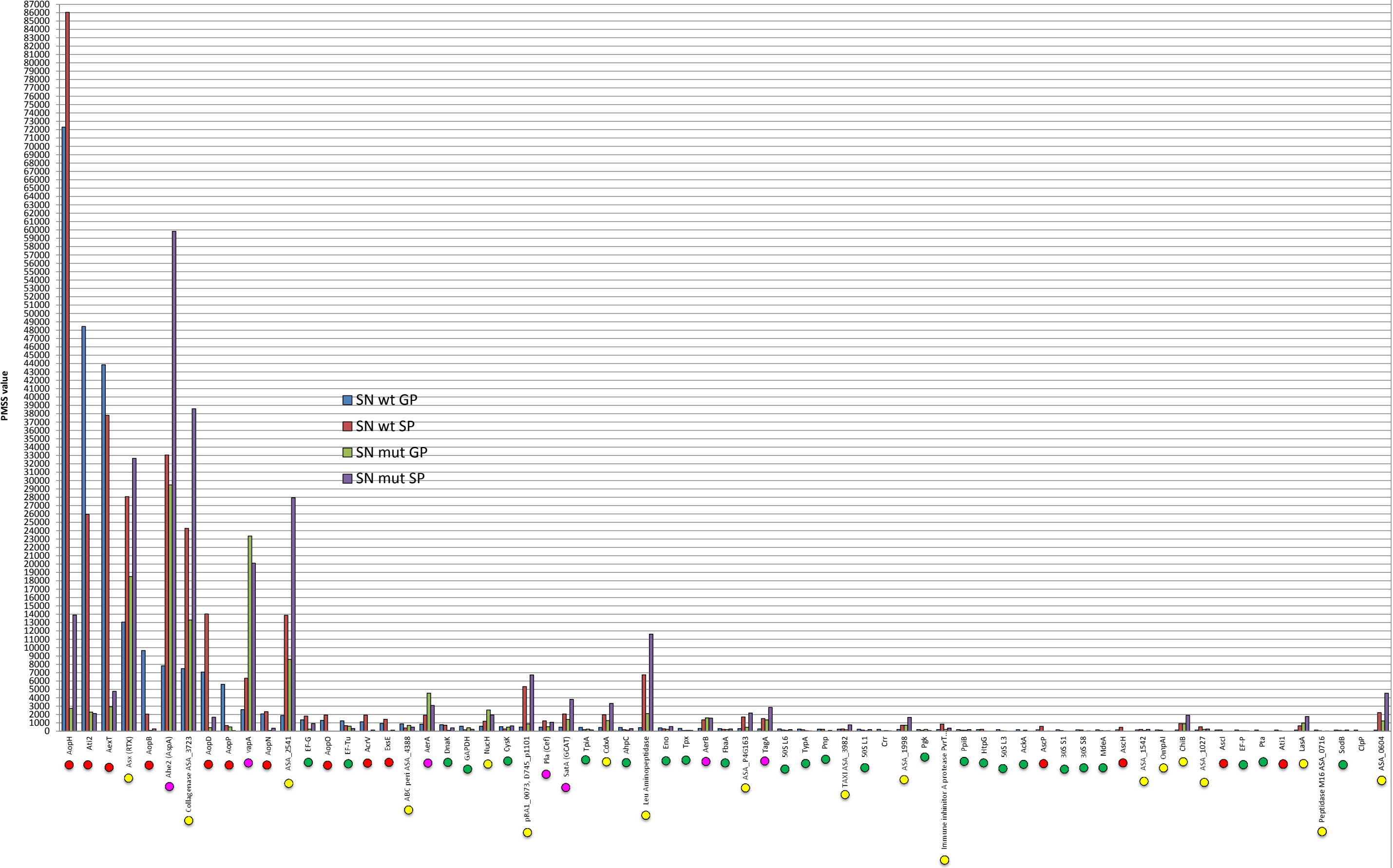

Supplement: Additional file 2 — Figure: most abundant proteins in A. salmonicida supernatants of wt and mutant strains in GP and SP. The diagram represents the most abundant proteins secreted by A. salmonicida (in decreasing order of PMSS values in wt SN during GP). Below the name of the protein circles represent T3SS components (red), other virulence factors (toxins, enzymes and adhesins) (pink), putative secondary virulence factors (yellow) and cytoplasmic proteins with putative moonlighting activity (green). [file 1477-5956-11-42-S2.pdf]

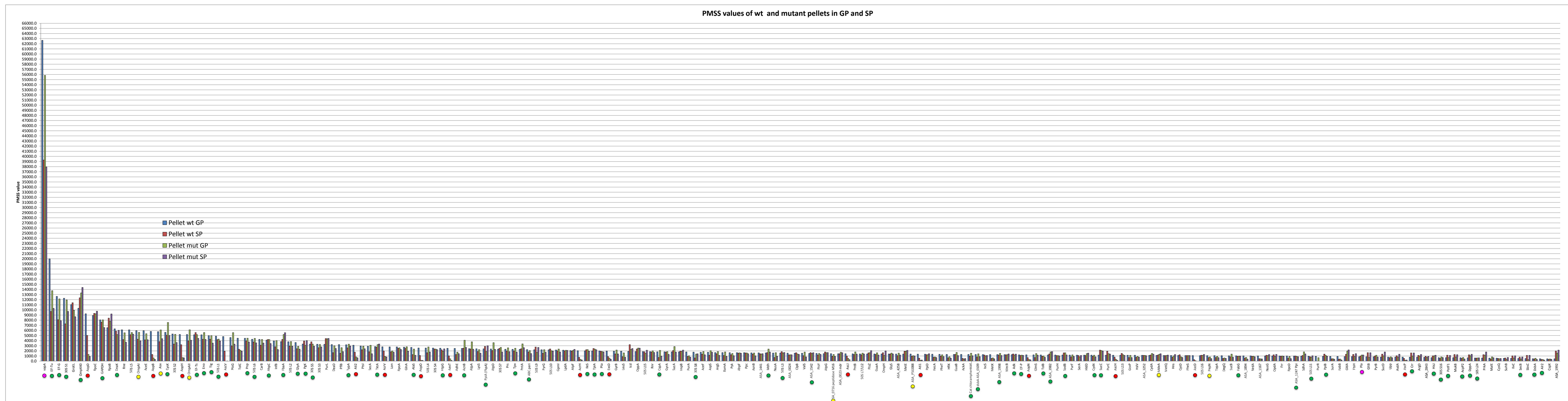

Supplement: Additional file 3 — Figure: most abundant proteins in A. salmonicida pellets of wt and mutant strains in GP and SP. The diagram represents the most abundant proteins detected in A. salmonicida pellets (in decreasing order of PMSS values in wt pellet during GP). Below the name of the protein circles represent T3SS components (red), other virulence factors (toxins, enzymes and adhesins) (pink), putative secondary virulence factors (yellow) and secreted cytoplasmic proteins with putative moonlighting activity (green). [file 1477-5956-11-42-S3.pdf]
