# Supplementary material for: The Aeromonas salmonicida subsp. salmonicida exoproteome: determination of the complete repertoire of Type-Three Secretion System effectors and identification of other virulence factors
Source: Proteome Sci. 2013 Sep 27;11:42. doi: 10.1186/1477-5956-11-42 (PMC3852671; doi:10.1186/1477-5956-11-42)
Supplement: Additional file 4 — Figure: confirmation by western blotting of the difference in quantity observed between the pellets of the wt and the ΔascV mutant in the GP for AopD, AcrV and AexT. [file 1477-5956-11-42-S4.pdf]

<u>Confirmation by western blotting of the difference in quantity observed between the pellets of the wt and the *ΔascV* mutant in the GP</u>

SDS-PAGE gels 15%

<u>Polyclonal antibodies:</u> anti-AopD | anti-AcrV | anti-ExoS (AexT)

<u>Pellets from the GP:</u> wt *ΔascV* | wt *ΔascV* | wt *ΔascV*

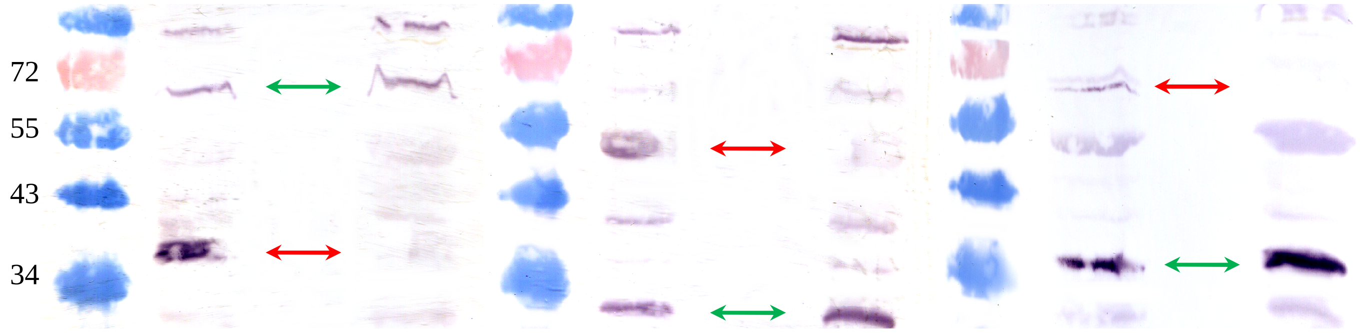

Red arrows show the signal for AopD, AcrV and AexT

Green arrows show nonspecific bands that were as much expressed in wt and *ΔascV* pellets confirming that same amounts of bacteria were loaded in the gel.
