# Supplementary material for: The Aeromonas salmonicida subsp. salmonicida exoproteome: determination of the complete repertoire of Type-Three Secretion System effectors and identification of other virulence factors
Source: Proteome Sci. 2013 Sep 27;11:42. doi: 10.1186/1477-5956-11-42 (PMC3852671; doi:10.1186/1477-5956-11-42)
Supplement: Additional file 5 — Table: A. salmonicida secreted proteins that have homologues in other bacteria with a putative role in virulence. [file 1477-5956-11-42-S5.doc]

| **Locus** | | **Name** | **Functions** | **Reference for *A. salmonicida*** | **Homologues in other bacteria with putative role in virulence** | **Reference** |
| --- | --- | --- | --- | --- | --- | --- |
| **Proteins without a peptide signal for secretion and oversecreted in wt SNs** | | | | | | |
| ASA_0427 | AcnB | Aconitase | - | - | AcnB of *Xanthomonas* | [1] |
| ASA_1442 | WecB | UDP-N-acetylglucosamine 2-epimerase | - | - | WecB of *Yersinia pestis* | [2] |
| **Proteins with a peptide signal for Sec-dependent secretion and oversecreted in wt SNs** | | | | | | |
| ASA_1267 | OmpAI | Outer membrane protein | - | - |  |  |
| ASA_1544 | OmpK40 | Outer membrane protein | C1q-binding 40 kDa porin | [3] | OmpA of *Escherichia coli*  OmpA of *Yersinia pestis*  Omp38 of *Acinetobacter baumannii* | [4-7] |
| ASA_0330 | DegQ | Periplasmic trypsin-like serine protease | - | - | HtrA of *Chlamydia* | [8,9] |
| ASA_0716 | - | Insulinase | zinc-dependent peptidase M16 | - | Insulinase of *Vibrio vulnificus* | [10] |
| ASA_0852 | - | Putative OM lipoprotein | - | - | - | [11] |
| **Well-described secreted virulence factors detected in equal quantity between wt (highly virulent) and *ΔascV* mutant (non virulent) SNs** | | | | | | |
| ASA_1438 | VapA | tetragonal surface virulence array protein | S-layer protein  Binding of laminin and fibronectin | [12,13] | Only in *Vibrio* sp. | - |
| ASA_3906 | AerA | Aerolysin A | Pore-forming toxin | - | Cytolytic/cytotoxic enterotoxin Act of *A. hydrophila*  VS4 vibrioaerolysin of *Vibrio splendidus* | [14,15] |
| ASA_2854 | AerB | Aerolysin B | Hemolysin, leukocidin and ricin domains | - | b-haemolysin or HlyA of *A. hydrophila*  Vah1 haemolysin of *Vibrio anguillarum* | [16-20] |
| ASA_0509 | SatA, GCAT | Esterase | Glycerophospholipid-cholesterol acyltransferase | [21] | GCAT of *A. hydrophila*  PlaA Legionella pneumophila | [19,22] |
| ASA_4288 | PlaA1 | Phospholipase A1 | - | - | Cytotonic enterotoxin Alt of *A. hydrophila* | [23] |
| ASA_0635 | PlaC | Phospholipase C | - | - | PlaC of mesophilic *Aeromonas* sp. | [24] |
| ASA_3321 | TagA | Metalloprotease/mucinase ToxR-regulated lipoprotein | Metalloprotease/mucinase | - | TagA of *A. hydrophila* and *V. cholerae*  StcE (secreted protease of complement C1-esterase inhibitor [C1-INH]) of enterohemorrhagic *E. coli* | [25-27] |
| ASA_2540 | Ahe2, AspA | Serine protease | Immunosuppressive protease | [21,28] | Kexin-like serine protease ASP of *A. sobria* or *V. parahaemolyticus* | [29,30] |
| ASA_0604 | - | Chitin/ N-acetylglucosamine-binding protein | - | - | GbpA of *Vibrio cholerae* lmo2467 of *Listeria monocytogenes*  CbpD of *P. aeruginosa* | [29,31-33] |
| ASA_1199 | Nuc | Endonuclease | - | - | EndA of *Shewanella oneidensis* | [34,35] |
| ASA_3475 | eno | Enolase | - | - | Enolase of *A. hydrophila* | [36] |
| ASA_3132 | PepO | Outer membrane endopeptidase | Activates human big endothelin-3 in vitro and induces skin ulcer in goldfish | - | PepO of *A. hydrophila* | [37] |
| **Putative secreted virulence factors detected in equal quantity between wt (highly virulent) and *ΔascV* mutant (non virulent) SNs** | | | | | | |
| ASA_0826 | Asx | RTX (repeats in toxin) exoprotein | Adhesin? | - | Haemagglutinating RTX protein [BCAM2143] of *Burkholderia cenocepacia*  TosA adhesin of uropathogenic *E. coli*  VVA0331 of *Vibrio vulnificus*  Biofilm-promoting factor A of *Shewanella* *oneidensis*  PnxIIIA of *Pasteurella pneumotropica* | [38-42] |
| ASA_3723 | - | microbial collagenase | - | - | Collagenase A of *Clostridium perfringens* | [43,44] |
| ASA_2206 | NucH | Nuclease | - | - | ExeM and ExeS nucleases of *Shewanella oneidensis*  Dns of *Vibrio cholera*  SpnA of *Streptococcus pyogenes* | [45-47] |
| ASA_3073 | - | Leucine aminopeptidase | - | - | LapA and LapB aminopeptidases of *Legionella pneumophila* | [48] |
| ASA_0873 | CdxA | Chitinase | - | - | Lmo0105 ChiB of *Listeria monocytogenes* | [32] |
| ASA_3982 | TAXI | TRAP-associated extracytoplasmic immunogenic | Solute receptor of a TRAP transporter | - | - | [49] |
| ASA_0849 | PrtV | Immune inhibitor A | Metalloprotease | - | Virulence metalloproteases InhA1, InhA2 and InhA3 of *Bacillus cereus*  Vsm of *Vibrio splendidus* | [50,51] |
| ASA_1287 | LasA | Metalloprotease | - | - | Staphylolysin LasA of *Pseudomonas aeruginosa* | [52] |
| ASA_1027 | - | LysM domain-containing protein | - | - | YkuD L,D-Transpeptidase of *Bacillus subtilis* | [53] |
| ASA_1998 | - | hypothetical GlyGly-CTERM protein | - | - | - | [54] |
| ASA_P4G031 | - | Micrococcal nuclease (SNase-like) | - | - | Nuclease SAV0815 of *Staphylococcus aureus* | [55] |
| ASA_2968 | Azu | Azurin | - | - | Azurin of *Pseudomonas aeruginosa* | [56] |
| ASA_3728 | FimD | Type I pilus subunit | - | - | FimD of *Bordetella pertussis* | [57] |
| ASA_1523 | - | Hemolysin? | - | - | Hemolysin of *A. hydrophila* | [58] |
| **Secreted virulence factors not detected in our work** | | | | | | |
| ASA_2003  ASA_2015 | Zot | Zonular Occludens Toxins | - | - | - | [59] |
| ASA_3440 | AhpB | Elastase | - | - | Vibriolysin and *P. aeruginosa* LasB | [60,61] |
| ASA_1660 | AsaP1 | Toxic endopeptidase | - | - | AsaP1 peptidase of *A. salmonicida* subsp *achromogenes* | [62] |
| ASA_2128 | btt | Insecticidal cytolytic delta-endotoxin | - | - | Cytolytic toxins of *Bacillus thuringiensis* | [63] |
| ASA_0628 | PulA | Pullulanase | - | - | *Streptococcus pyogenes* | [64] |

**References:**

1. Kirchberg J, Buttner D, Thiemer B, Sawers RG: **Aconitase B is required for optimal growth of *Xanthomonas campestris* pv. *vesicatoria* in pepper plants.** *Plos One* 2012, **7**.

2. Klein KA, Fukuto HS, Pelletier M, Romanov G, Grabenstein JP, Palmer LE, Ernst R, Bliska JB: **A transposon site hybridization screen identifies *galU* and *wecBC* as important for survival of *Yersinia pestis* in murine macrophages.** *J Bact* 2012, **194:**653-662.

3. Merino S, Vilches S, Canals R, Ramirez S, Tomas JM: **A C1q-binding 40 kDa porin from *Aeromonas salmonicida*: Cloning, sequencing, role in serum susceptibility and fish immunoprotection.** *Microb Pathog* 2005, **38:**227-237.

4. Maruvada R, Kim KS: **Extracellular loops of the *Eschericia coli* outer membrane protein A contribute to the pathogenesis of meningitis.** *J Infect Dis* 2011, **203:**131-140.

5. Bartra SS, Gong X, Lorica CD, Jain C, Naire MKM, Schifferli D, Qian LF, Li ZW, Plano GV, Schesser K: **The outer membrane protein A (OmpA) of *Yersinia pestis* promotes intracellular survival and virulence in mice.** *Microb Pathogenesis* 2012, **52:**41-46.

6. Choi CH, Lee EY, Lee YC, Park TI, Kim HJ, Hyun SH, Kim SA, Lee SK, Lee JC: **Outer membrane protein 38 of *Acinetobacter baumannii* localizes to the mitochondria and induces apoptosis of epithelial cells.** *Cell Microbiol* 2005, **7:**1127-1138.

7. Choi CH, Hyun SH, Lee JY, Lee JS, Lee YS, Kim SA, Chae JP, Yoo SM, Lee JC: ***Acinetobacter baumannii* outer membrane protein A targets the nucleus and induces cytotoxicity.** *Cell Microbiol* 2008, **10:**309-319.

8. Zijnge V, Kieselbach T, Oscarsson J: **Proteomics of protein secretion by *Aggregatibacter actinomycetemcomitans*.** *Plos One* 2012, **7**.

9. Wu X, Lei L, Gong S, Chen D, Flores R, Zhong G: **The chlamydial periplasmic stress response serine protease cHtrA is secreted into host cell cytosol.** *BMC Microbiol* 2011, **11:**87.

10. Ryu Y, Kim YJ, Kim YR, Seok YJ: **Expression of *Vibrio vulnificus* insulin-degrading enzyme is regulated by the cAMP-CRP complex.** *Microbiology - SGM* 2012, **158:**1294-1303.

11. Kenedy MR, Lenhart TR, Akins DR: **The role of *Borrelia burgdorferi* outer surface proteins.** *FEMS Immunol Med Mic* 2012, **66:**1-19.

12. Chu S, Cavaignac S, Feutrier J, Phipps BM, Kostrzynska M, Kay WW, Trust TJ: **Structure of the tetragonal surface virulence array protein and gene of *Aeromonas salmonicida*.** *J Biol Chem* 1991, **266:**15258-15265.

13. Doig P, Emody L, Trust TJ: **Binding of laminin and fibronectin by the trypsin-resistant major structural domain of the crystalline virulence surface array protein of *Aeromonas salmonicida*.** *J Biol Chem* 1992, **267:**43-49.

14. Bucker R, Krug SM, Rosenthal R, Gunzel D, Fromm A, Zeitz M, Chakraborty T, Fromm M, Epple HJ, Schulzke JD: **Aerolysin from *Aeromonas hydrophila* perturbs tight junction integrity and cell lesion repair in intestinal epithelial HT-29/B6 cells.** *J Infect Dis* 2011, **204:**1283-1292.

15. MacPherson HL, Bergh O, Birkbeck TH: **An aerolysin-like enterotoxin from *Vibrio splendidus* may be involved in intestinal tract damage and mortalities in turbot, *Scophthalmus maximus* (L.), and cod, *Gadus morhua* L., *larvae*.** *J Fish Dis* 2012, **35:**153-167.

16. Zhang YL, Ong CT, Leung KY: **Molecular analysis of genetic differences between virulent and avirulent strains of *Aeromonas hydrophila* isolated from diseased fish.** *Microbiology -UK* 2000, **146:**999-1009.

17. Wong CYF, Heuzenroeder MW, Flower RLP: **Inactivation of two haemolytic toxin genes in *Aeromonas hydrophila* attenuates virulence in a suckling mouse model.** *Microbiology -UK* 1998, **144:**291-298.

18. Yu HB, Zhang YL, Lau YL, Yao F, Vilches S, Merino S, Tomas JM, Howard SP, Leung KY: **Identification and characterization of putative virulence genes and gene clusters in *Aeromonas hydrophila* PPD134/91.** *Appl Environ Microbiol* 2005, **71:**4469-4477.

19. Yu HB, Kaur R, Lim SM, Wang XH, Leung KY: **Characterization of extracellular proteins produced by *Aeromonas hydrophila* AH-1.** *Proteomics* 2007, **7:**436-449.

20. Li L, Rock JL, Nelson DR: **Identification and characterization of a repeat-in-toxin gene cluster in *Vibrio anguillarum*.** *Infect Immun* 2008, **76:**2620-2632.

21. Vipond R, Bricknell IR, Durant E, Bowden TJ, Ellis AE, Smith M, MacIntyre S: **Defined deletion mutants demonstrate that the major secreted toxins are not essential for the virulence of *Aeromonas salmonicida*.** *Infect Immun* 1998, **66:**1990-1998.

22. Flieger A, Neumeister B, Cianciotto NP: **Characterization of the gene encoding the major secreted lysophospholipase A of *Legionella pneumophila* and its role in detoxification of lysophosphatidylcholine.** *Infect Immun* 2002, **70:**6094-6106.

23. Chopra AK, Peterson JW, Xu XJ, Coppenhaver DH, Houston CW: **Molecular and biochemical characterization of a heat-labile cytotonic enterotoxin from *Aeromonas hydrophila*.** *Microb Pathog* 1996, **21:**357-377.

24. Merino S, Aguilar A, Nogueras MM, Regue M, Swift S, Tomas JM: **Cloning, sequencing, and role in virulence of two phospholipases (A1 and C) from mesophilic *Aeromonas* sp serogroup O : 34.** *Infect Immun* 1999, **67:**4008-4013.

25. Pillai L, Sha J, Erova TE, Fadl AA, Khajanchi BK, Chopra AK: **Molecular and functional characterization of a ToxR-regulated lipoprotein from a clinical isolate of *Aeromonas hydrophila*.** *Infect Immun* 2006, **74:**3742-3755.

26. Szabady RL, Yanta JH, Halladin DK, Schofield MJ, Welch RA: **TagA is a secreted protease of *Vibrio cholerae* that specifically cleaves mucin glycoproteins.** *Microbiology - SGM* 2011, **157:**516-525.

27. Yu AC, Worrall LJ, Strynadka NC: **Structural insight into the bacterial mucinase StcE essential to adhesion and immune evasion during enterohemorrhagic *E. coli* infection.** *Structure* 2012, **20:**707-717.

28. Hussain I, Mackie C, Cox D, Alderson R, Birkbeck TH: **Suppression of the humoral immune response of Atlantic salmon, *Salmo salar* L. by the 64 kDa serine protease of *Aeromonas salmonicida*.** *Fish Shellfish Immun* 2000, **10:**359-373.

29. Mao ZJ, Yu L, You ZQ, Wei YW, Liu Y: **Cloning, expression and immunogenicty analysis of five outer membrane proteins of *Vibrio parahaemolyticus* zj2003.** *Fish Shellfish Immun* 2007, **23:**567-575.

30. Kobayashi H, Utsunomiya H, Yamanaka H, Sei Y, Katunuma N, Okamoto K, Tsuge H: **Structural basis for the kexin-like serine protease from *Aeromonas sobria* as sepsis-causing factor.** *J Biol Chem* 2009, **284:**27655-27663.

31. Wong E, Vaaje-Kolstad G, Ghosh A, Hurtado-Guerrero R, Konarev PV, Ibrahim AFM, Svergun DI, Eijsink VGH, Chatterjee NS, van Aalten DMF: **The *Vibrio cholerae* colonization factor GbpA possesses a modular structure that governs binding to different host surfaces.** *PLoS Pathog* 2012, **8**.

32. Chaudhuri S, Bruno JC, Alonzo F, Xayarath B, Cianciotto NP, Freitag NE: **Contribution of chitinases to *Listeria monocytogenes* pathogenesis.** *Appl Environ Microbiol* 2010, **76:**7302-7305.

33. Folders J, Tommassen J, van Loon LC, Bitter W: **Identification of a chitin-binding protein secreted by *Pseudomonas aeruginosa*.** *J Bact* 2000, **182:**1257-1263.

34. Heun M, Binnenkade L, Kreienbaum M, Thormann KM: **Functional specificity of extracellular nucleases of *Shewanella oneidensis* MR-1.** *Appl Environ Microbiol* 2012, **78:**4400-4411.

35. Nam IY, Joh K: **Rapid detection of virulence factors of *Aeromonas* isolated from a trout farm by hexaplex-PCR.** *J Microbiol* 2007, **45:**297-304.

36. Sha J, Erova TE, Alyea RA, Wang SF, Olano JP, Pancholi V, Chopra AK: **Surface-expressed enolase contributes to the pathogenesis of clinical isolate SSU of *Aeromonas hydrophila*.** *J Bact* 2009, **191:**3095-3107.

37. Abolghait SK, Akeda Y, Kodama T, Cantarelli VV, Iida T, Honda T: ***Aeromonas hydrophila* PepO outer membrane endopeptidase activates human big endothelin-3 in vitro and induces skin ulcer in goldfish (*Carassius auratus*).** *Vet Microbiol* 2010, **145:**113-121.

38. Whitby PW, VanWagoner TM, Taylor AA, Seale TW, Morton DJ, Lipuma JJ, Stull TL: **Identification of an RTX determinant of *Burkholderia cenocepacia* J2315 by subtractive hybridization.** *J Med Microbiol* 2006, **55:**11-21.

39. Vigil PD, Wiles TJ, Engstrom MD, Prasov L, Mulvey MA, Mobley HLT: **The repeat-in-toxin family member TosA mediates adherence of uropathogenic *Escherichia coli* and survival during bacteremia.** *Infect Immun* 2012, **80:**493-505.

40. Sasaki H, Ishikawa H, Sato T, Sekiguchi S, Amao H, Kawamoto E, Matsumoto T, Shirama K: **Molecular and virulence characteristics of an outer membrane-associated RTX exoprotein in *Pasteurella pneumotropica*.** *BMC Microbiol* 2011, **11**.

41. Chou LF, Peng HL, Yang YC, Kuo MC, Chang HY: **Localization and characterization of VVA0331, a 489-kDa RTX-like protein, in *Vibrio vulnificus* YJ016.** *Arch Microbiol* 2009, **191:**441-450.

42. Theunissen S, De Smet L, Dansercoer A, Motte B, Coenye T, Van Beeumen JJ, Devreese B, Savvides SN, Vergauwen B: **The 285 kDa Bap/RTX hybrid cell surface protein (SO4317) of *Shewanella oneidensis* MR-1 is a key mediator of biofilm formation.** *Res Microbiol* 2010, **161:**144-152.

43. Awad MM, Ellemor DM, Bryant AE, Matsushita O, Boyd RL, Stevens DL, Emmins JJ, Rood JI: **Construction and virulence testing of a collagenase mutant of *Clostridium perfringens*.** *Microb Pathog* 2000, **28:**107-117.

44. Shi L, Ermis R, Garcia A, Telgenhoff D, Aust D: **Degradation of human collagen isoforms by *Clostridium* collagenase and the effects of degradation products on cell migration.** *Int Wound J* 2010, **7:**87-95.

45. Godeke J, Heun M, Bubendorfer S, Paul K, Thormann KM: **Roles of two *Shewanella oneidensis* MR-1 extracellular endonucleases.** *Appl Environ Microbiol* 2011, **77:**5342-5351.

46. Chang A, Khemlani A, Kang H, Proft T: **Functional analysis of *Streptococcus pyogenes* nuclease A (SpnA), a novel group A streptococcal virulence factor.** *Mol Microbiol* 2011, **79:**1629-1642.

47. Blokesch M, Schoolnik GK: **The extracellular nuclease Dns and its role in natural transformation of *Vibrio cholerae*.** *J Bact* 2008, **190:**7232-7240.

48. Rossier O, Dao J, Cianciotto NP: **The type II secretion system of *Legionella pneumophila* elaborates two aminopeptidases, as well as a metalloprotease that contributes to differential infection among protozoan hosts.** *Appl Environ Microbiol* 2008, **74:**753-761.

49. Mulligan C, Fischer M, Thomas GH: **Tripartite ATP-independent periplasmic (TRAP) transporters in bacteria and archaea.** *FEMS Microbiol Rev* 2011, **35:**68-86.

50. Guillemet E, Cadot C, Tran SL, Guinebretiere MH, Lereclus D, Ramarao N: **The InhA metalloproteases of *Bacillus cereus* contribute concomitantly to virulence.** *J Bact* 2010, **192:**286-294.

51. Binesse J, Delsert C, Saulnier D, Champomier-Verges MC, Zagorec M, Munier-Lehmann I, Mazel D, Le Roux F: **Metalloprotease Vsm is the major determinant of toxicity for extracellular products of *Vibrio splendidus*.** *Appl Environ Microbiol* 2008, **74:**7108-7117.

52. Spencer J, Murphy LM, Conners R, Sessions RB, Gamblin SJ: **Crystal structure of the LasA virulence factor from *Pseudomonas aeruginosa*: Substrate specificity and mechanism of M23 metallopeptidases.** *J Mol Biol* 2010, **396:**908-923.

53. Bielnicki J, Devedjiev Y, Derewenda U, Dauter Z, Joachimiak A, Derewenda ZS: ***B-subtilis* *ykuD* protein at 2.0 A resolution: insights into the structure and function of a novel, ubiquitous family of bacterial enzymes.** *Proteins* 2006, **62:**144-151.

54. Haft DH, Varghese N: **GlyGly-CTERM and rhombosortase: a C-terminal protein processing signal in a many-to-one pairing with a rhomboid family intramembrane serine protease.** *Plos One* 2011, **6**.

55. Berends ETM, Horswill AR, Haste NM, Monestier M, Nizet V, Kockritz-Blickwede M: **Nuclease expression by *Staphylococcus aureus* facilitates escape from neutrophil extracellular traps.** *J Innate Immun* 2010, **2:**576-586.

56. Goto M, Yamada G, Kimbara K, Horner J, Newcomb M, Das Gupta TK, Chakrabarty AM: **Induction of apoptosis in macrophages by *Pseudomonas aeruginosa* azurin: tumour-suppressor protein p53 and reactive oxygen species, but not redox activity, as critical elements in cytotoxicity.** *Mol Microbiol* 2003, **47:**549-559.

57. Geuijen CAW, Willems RJL, Bongaerts M, Top J, Gielen H, Mooi FR: **Role of the *Bordetella pertussis* minor fimbrial subunit, FimD, in colonization of the mouse respiratory tract.** *Infect Immun* 1997, **65:**4222-4228.

58. Erova TE, Sha J, Horneman AJ, Borchardt MA, Khajanchi BK, Fadl AA, Chopra AK: **Identification of a new hemolysin from diarrheal isolate SSU of *Aeromonas hydrophila*.** *FEMS Microbiol Let* 2007, **275:**301-311.

59. Fasano A: **Regulation of intercellular tight junctions by Zonula occludens toxin and its eukaryotic analogue zonulin.** *Epithelial Transport and Barrier Function* 2000, **915:**214-222.

60. Iqbal A, Azim MK, Hashmi N, Ali SA, Musharraf SG: **Structural characterization of metalloprotease vibriolysin of cholera pathogen *Vibrio cholerae*.** *Protein Pept Lett* 2011, **18:**287-294.

61. Cowell BA, Twining SS, Hobden JA, Kwong MSF, Fleiszig SMJ: **Mutation of lasA and lasB reduces *Pseudomonas aeruginosa* invasion of epithelial cells.** *Microbiology - SGM* 2003, **149:**2291-2299.

62. Arnadottir H, Hvanndal I, Andresdottir V, Burr SE, Frey J, Gudmundsdottir BK: **The AsaP1 peptidase of *Aeromonas salmonicida* subsp *achromogenes* is a highly conserved deuterolysin metalloprotease (family M35) and a major virulence factor.** *J Bact* 2009, **191:**403-410.

63. Cohen S, Albeck S, Ben Dov E, Cahan R, Firer M, Zaritsky A, Dym O: **Cyt1Aa toxin: crystal structure reveals implications for its membrane-perforating function.** *J Mol Biol* 2011, **413:**804-814.

64. Hytonen J, Haataja S, Finne J: ***Streptococcus pyogenes* glycoprotein-binding strepadhesin activity is mediated by a surface-associated carbohydrate-degrading enzyme, pullulanase.** *Infect Immun* 2003, **71:**784-793.
